# Low Level Engraftment and Improvement following a Single Colonoscopic Administration of Fecal Microbiota to Patients with Ulcerative Colitis

**DOI:** 10.1371/journal.pone.0133925

**Published:** 2015-08-19

**Authors:** Christopher J. Damman, Mitchell J. Brittnacher, Maria Westerhoff, Hillary S. Hayden, Matthew Radey, Kyle R. Hager, Sara R. Marquis, Samuel I. Miller, Timothy L. Zisman

**Affiliations:** 1 Department of Medicine, Division of Gastroenterology, University of Washington, Seattle, Washington, 98195, United States of America; 2 Department of Microbiology, University of Washington, Seattle, Washington, 98195, United States of America; 3 Department of Anatomic Pathology, University of Washington, Seattle, Washington, 98195, United States of America; University Hospital Llandough, UNITED KINGDOM

## Abstract

**Objective:**

Fecal microbiota transplantation (FMT) is an investigational treatment for diseases thought to involve alterations in the intestinal microbiota including ulcerative colitis (UC). Case reports have described therapeutic benefit of FMT in patients with UC, possibly due to changes in the microbiota. We measured the degree to which the transplanted microbiota engraft following FMT in patients with UC using a donor similarity index (DSI).

**Methods:**

Seven patients with mild to moderate UC (UC disease activity index scores 3–10) received a single colonoscopic administration of FMT. Metagenomic sequence data from stool were analyzed using an alignment-free comparison tool, to measure the DSI, and a phylogenetic analysis tool, to characterize taxonomic changes. Clinical, endoscopic, histologic, and fecal calprotectin outcome measures were also collected.

**Results:**

One of 5 patients from whom sequencing data were available achieved the primary endpoint of 50% donor similarity at week 4; an additional 2 patients achieved 40% donor similarity. One patient with 40% donor similarity achieved clinical and histologic remission 1 month after FMT. However, these were lost by 2−3 months, and loss correlated with a decrease in DSI. The remaining patients did not demonstrate clinical response or remission. Histology scores improved in all but 1 patient. No patients remained in remission at 3 months after FMT.

**Conclusions:**

Following a single colonoscopic fecal transplant, a DSI of 40-50% is achieved in about two-thirds of recipients. This level of engraftment correlated with a temporary clinical improvement in only 1/5 patients. Larger sample sizes could further validate this method for measuring engraftment, and changes in transplant frequency or method might improve microbiota engraftment and efficacy.

**Trial Registration:**

ClinicalTrials.gov NCT01742754

## Introduction

Ulcerative colitis (UC) is a chronic relapsing inflammatory disorder of the colon affecting 0.24% of the population.[1] Current medical treatments for UC include aminosalicylates, thiopurines, corticosteroids, tumor necrosis factor-modulating agents, and other immunosuppressives.[2] These treatments are often limited by actual or perceived risk of side effects, including opportunistic infections, lymphoma, and a lack of response in many patients. Indeed, up to twenty-five percent of patients with UC ultimately require colectomy, most commonly for medically refractory disease, highlighting the need for new medical therapies.[3]

In recent years, with the advent of culture-independent sequencing technologies, there have been mounting efforts to understand the role that endogenous gut microbes play in UC and develop new microbial-based therapies. Several studies show decreases in normal anaerobic flora (e.g. Clostridial clusters IV and XIVa and *Bacteroides ssp*.) with reciprocal increases in pathobionts (e.g. Proteobacteria).[4–6] The extent to which alterations in the microbiome are a cause or result of inflammation remains unknown. Studies using antibiotics[7] and probiotics[8] for UC have had some success suggesting that alteration of the microbiota may be a successful strategy to achieve disease control in some patients.

Unlike antibiotics that alter the microbiota through further disruption of the normal flora, FMT offers the hope of repleting healthy commensal species and restoring eubiosis. Over 300 case reports and a placebo-controlled trial have demonstrated that FMT is safe and efficacious in the treatment of *Clostridium difficile*-associated diarrhea. [9,10] More recently, FMT has been studied in some individuals with UC with modest results.[11,12] Several case reports of FMT have resulted in sustained remission, [13–15] and more recently two randomized controlled trials have shown mixed results.[16,17] The role of microbiota engraftment in possibly inducing remission is unclear. We therefore undertook a study to evaluate the effect of a single colonoscopic fecal transplant in mildly to moderately active UC using metagenomic sequencing data to calculate the donor similarity index (DSI) as a measure of engraftment.

## Materials and Methods

### Study Oversight

This study was reviewed and initially approved by the University of Washington institutional review board in April 2012 (protocol number 41454). Several modifications were made to the protocol prior to enrolling patients and the final protocol was approved October 2012.(S1 Protocol) At the time the study was initiated, fecal microbiota therapy did not fall into the category of a drug or biologic, and given that our primary aim was to evaluate engraftment rather than clinical outcomes, it was unclear whether registration with www.clinicaltrials.gov was necessary. Nonetheless, within 30 days of recruiting the first patient, the study was registered at *www*.*clinicaltrials*.*gov* (registration number NCT01742754). The authors confirm that all related trials for this intervention will be registered with www.clinicaltrials.gov. Written informed consent was obtained from all study subjects including both patients and stool donors. Per the IRB submitted protocol we intended to enroll 10 patients and 10 donors. Prior to completion of the study, new FDA regulations came out requiring an IND for FMT studies. We terminated the study early and have since applied for an IND under a different protocol.

### Study Design

This is a prospective, open-label, uncontrolled, single-center pilot study conducted at the University of Washington’s Digestive Disease Center. Subjects were enrolled between October 2012 and May of 2013. Patients received a single colonoscopic FMT and were followed for up to three months after intervention. If a patient developed a flare prior to three months, a last fecal sample was collected at this time.

### Patient Selection

Patients were recruited from the University of Washington’s outpatient inflammatory bowel disease clinic and were at least 18 years of age with mildly to moderately active UC as determined by a UC disease activity index (UCDAI) score of 3 to 10.[18] All patients underwent a baseline screening colonoscopy to confirm their diagnosis of UC prior to undergoing FMT. Exclusion criteria included antibiotic, biologic, or immunomodulatory therapy (thiopurines or methotrexate) within the last 3 months. Patients taking corticosteroid therapy or probiotics required a washout period of 2 weeks. With the exception of the above medications, patients were instructed to continue their current medications including 5-aminosalicylates during the duration of the study. Patients who tested positive for *C*. *difficile* by polymerase chain reaction (PCR) were excluded.

### Donor Selection

Donors were chosen by each recipient and had to be at least 18 years of age. They were screened for infection risks using a modified American Association of Blood Banks Donor History Questionnaire (S1 Questionnaire). Additional exclusion criteria included history of irritable bowel syndrome, inflammatory bowel disease, gastrointestinal malignancy, gastrointestinal polyps, use of antibiotics in the preceding 3 months, immunosuppressive medications, systemic antineoplastic agents, and recent ingestion of a potential allergen. Donors were also screened for infectious agents according to the FMT workgroup recommendations.[9] All donors were screened for HIV, HAV, HBV, HCV, and *C*. *difficile*. Donors who were not intimately involved with the subject were additionally screened for stool enterics, fecal *Giardia*, *Cryptosporidium*, *Cyclospora*, *Isospora*, ova and parasites, and syphilis.

### Donor Stool Preparation and Administration

Prior to FMT, patients underwent a bowel preparation with 4L of GoLYTELY (Braintree Laboratories, Inc. Braintree, MA) or an equivalent generic polyethylene glycol and electrolyte solution. No antibiotics were administered for the purpose of this study. Donor stool was produced fresh on the day of transplant and in all cases was prepared and transplanted within 6 hours. In the majority of cases, stool was transplanted within 2 to 3 hours. Donor stool was weighed and then diluted with 2-3mL of 0.9% normal saline per gram of stool, depending on the consistency of the donated stool. Mixing was performed manually using a tongue depressor. The stool mixture was then filtered through gauze and aspirated into 60 cc syringes. A total of 175 to 290 cc of this stool mixture was then administered to the terminal ileum and right colon through the working channel of a colonoscope.

### Endpoints

The primary endpoint was engraftment of donor stool at 4 weeks post-FMT defined as a DSI of greater than 50% indicating that the microbiota resembled the donor more than the recipient. The original endpoint in the IRB submitted protocol was based on 16S rRNA sequencing and UniFrac distances. After submission of the protocol and recruitment of patients, but before any sequencing was performed the protocol was amended to perform metagenomic sequencing with alignment-free sequence similarity analysis using Compareads[19] to take advantage of advancements in the field and provide an alternative measure of engraftment. Secondary endpoints included a DSI score of greater than 50% at 1 week and 12 weeks post-FMT, clinical response, clinical remission, a decrease in fecal calprotectin, and histologic improvement at week 4. We defined response as a decrease in total UCDAI score of 3 or more[18] and remission as a total UCDAI score of 2 or less with no individual subscore greater than 1 at 4 weeks after transplant. We also followed a 6-point UCDAI score (fecal blood and stool frequency) to provide non-invasive evaluation of clinical efficacy at time points before and after endoscopy. The 6-point partial UCDAI score has been shown to perform as well as the full UCDAI in assessing clinical response in UC.[20] Response in the 6-point UCDAI score was defined as a decrease of 2 or greater.

### Sample Collection

Donor stool was collected at about 1 week prior to FMT and again on the day of the transplant. Recipient stool was collected at two time points prior to transplant as well as at 1 week, 4 weeks and 12 weeks after transplant or at the time of clinical exacerbation anytime after week 4. Baseline samples (prior to FMT) were separated by at least 1 week and in most cases by greater than 1 month. Approximately 100–200 mg of stool was collected using a straw core technique[21] and placed into two different cryotubes: one containing 750cc of PowerBead Solution (MoBio, Carlsbad, CA) and another one that was empty. These samples were homogenized and frozen at -80°C within 30 minutes of processing. Biopsy samples were also collected from the patient during the initial colonoscopy (prior to FMT) and at week 4 during sigmoidoscopy. No bowel preparation was prescribed prior to the flexible sigmoidoscopy. Biopsy samples were placed in formalin for clinical processing and histological analysis.

### DNA Extraction

Bacterial genomic DNA was extracted using the PowerSoil DNA Isolation Kit (MoBio, Carlsbad, CA) with the some modifications. One g of 3 mm glass beads (Fisher Scientific, Pittsburgh, PA) was substituted for the garnet beads provided in the kit. Two additional incubation steps (65°C for 10 minutes and 95°C for 10 minutes) were inserted after the addition of solution C1 and prior to mechanical disruption (bead beating).

### Metagenomic Sequencing

Sequencing was performed on either the Illumina HiSeq 2000 or MiSeq platform. Sequencing libraries were constructed from genomic DNA using Illumina’s Nextera technology (Illumina, Inc., San Diego, CA). Briefly, DNA preparations were simultaneously fragmented and tagged with adapter oligomers. A limited-cycle PCR reaction amplified all tagged fragments and added: 1) index sequences (the dual indexing strategy uses two 8-base indices) to allow demultiplexing of sequence reads for pooled samples, and 2) sequencing primer sequences. Following PCR enrichment, libraries were denatured and hybridized via DNA/DNA binding of adaptors to existing features on a glass flow cell compatible with the Illumina sequencers. Sequencing was performed using well-established, ultra-high throughput methods. The HiSeq2000 produced approximately 200 million pairs of 93 bp reads per lane, and we generated 25–30 million raw read pairs per sample using 7-8-plex pools. The single lane of the MiSeq generated 10–20 million raw pairs of 150 bp reads per sample. These levels of sequencing provided sufficient depth, post-human and quality filtering as described below, to measure genera represented by greater than 0.2% abundance in the microbiota. The metagenomic shotgun sequence was deposited in the National Center for Biotechnology Information (NCBI) Sequence Read Archive (SRA) with BioProject accession number PRJNA285502.

### Sequence Quality Control and Analysis

Human DNA sequence was identified and removed using BMTagger[22] with the Hg-19 *Homo sapiens* reference genome. Duplicate reads were marked and removed using EstimateLibraryComplexity, part of the Picard tool package. Reads with ambiguous bases were trimmed from each end. Reads with Phred quality scores less than 6 over the first 80 (HiSeq) or 120 (MiSeq) bases of each read and reads shorter than 80 (HiSeq) or 120 (MiSeq) bases after trimming were removed. Samples with less than 10 million reads following filtering and Human sequence removal were not considered for further analysis. We determined relative species abundance from species specific maker genes using MetaPhlAn [23].

### Donor Similarity Index Calculation

The similarity between metagenomic samples was calculated using Compareads[19] with two *k*-mers of length 30 bases. The DSI for the longitudinal samples was calculated as the percent change in recipient to donor similarity relative to baseline similarity to donor. *S*_0_ is the pre-transplant similarity of the recipient to the donor (baseline sample), and *S*_*t*_ is the post-transplant similarity of the recipient to the donor at a given time point.

$$DSI=100\;\left(\frac{S_{t}-S_{0}}{100-S_{0}}\right),$$ (1)

### Fecal Calprotectin

Fecal Calprotectin was measured using the PhiCal Test (Calpro, Oslo, Norway; US distributer Genova Diagnostics, Ashville, NC).

### Histology

Biopsy samples targeting the most distal inflamed site within the rectum were placed in formalin, processed and reviewed by a gastroenterology-trained pathologist in a blinded fashion. Biopsies were scored based on the following scale: mild(1) = neutrophilic cryptitis, moderate(2) = crypt abscesses, and severe(3) = ulcers. A significant clinical response was defined as a change of 2 or more.

### Statistical Analysis

All statistical tests were performed with Excel (version 2011; Microsoft, Redmond, WA), GraphPad Prism software (version 6; GraphPad Software, La Jolla, CA), or MedCalc (version 15.4; MedCalc Software bvba, Ostend, Belgium). Sample diversity was measured before and after transplant to evaluate for any post transplant increases in diversity using the Shannon-Weiner diversity index described previously[24] in Excel. Comparisons in colitis scores and fecal calprotectin levels were made by repeated ANOVA in MedCalc. Comparisons of species abundance differences between donors and recipients were performed using a paired 2-tailed TTEST in Excel. Statistical correction for multiple testing was performed using the False Discovery Rate (FDR) method. P values of less than 0.05 were considered statistically significant.

## Results

### Patient and Donor Characteristics

**Fig 1:**
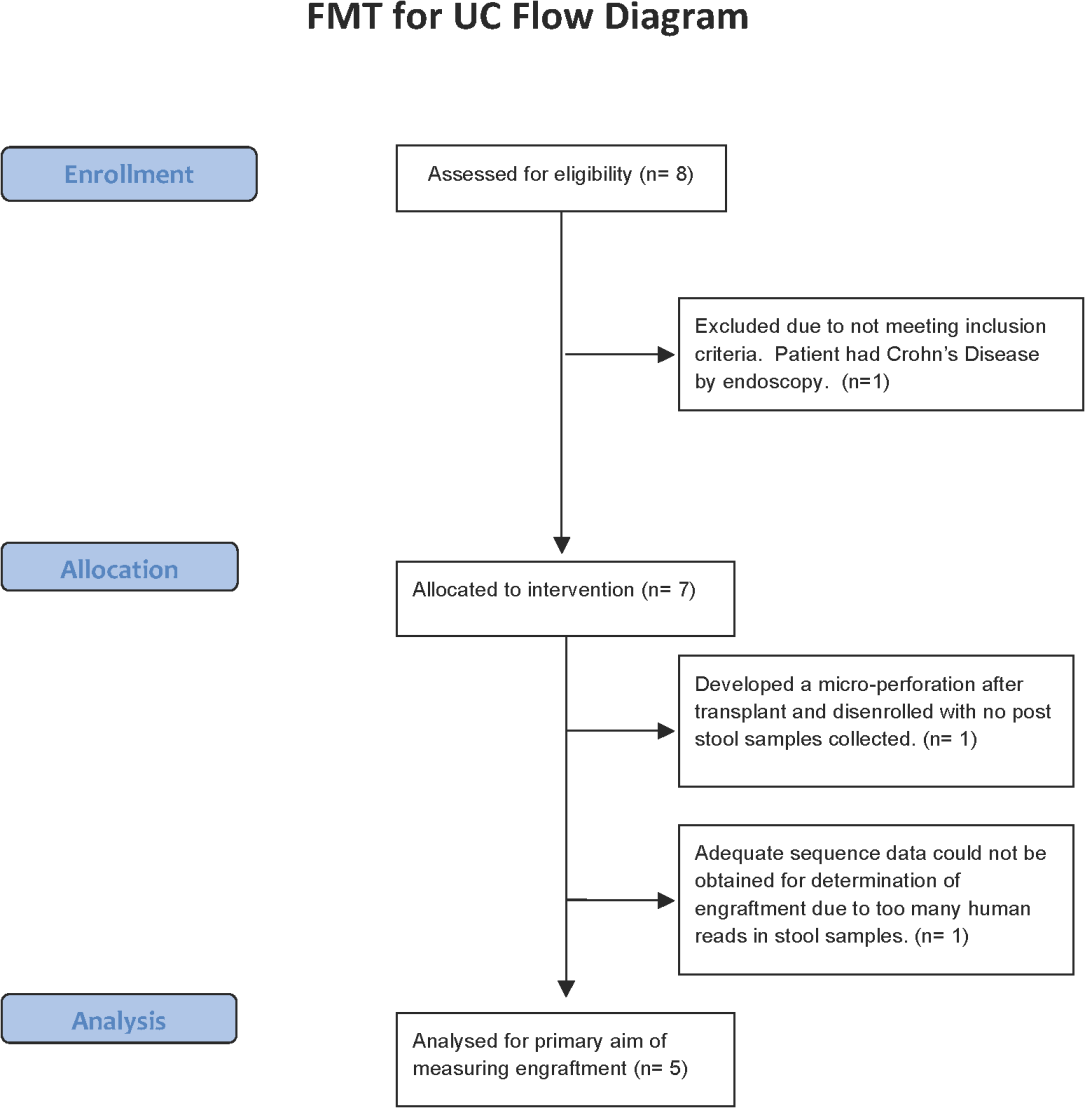
Flow diagram of patient recruitment.

There were eight patients enrolled in the study (Fig 1). The fourth patient on index colonoscopy was discovered to have Crohn’s disease and was dis-enrolled from the study without further clinical or microbiota analysis. The remaining seven patients with endoscopically confirmed UC underwent FMT (Table 1). The patients’ median age was 41, median disease duration was 17 years, median baseline UCDAI score was 7.6, and median fecal calprotectin level was 523 μg/g. Four patients had left-sided colitis and the remainder had pancolitis. Four patients had a history of extra-intestinal manifestations of UC including iritis/uveitis, pyostomatitis vegetans, aphthous stomatitis, and possible sacroiliitis. Three patients had a family history of UC. Patient three and patient five were mother and son respectively. Two patients were active smokers but were smoking at most 1 cigarette per day. All patients had previously received treatment with aminosalicylates. All donors were male (Table 2). The average age of the donors was 41 and the average BMI was 24. Four of the 7 donors lived with their respective recipient. The donor for patients 3 and 5 was the same individual (donor 3 and donor 3’).

**Table 1:** Patient Demographics and Disease Characteristics.

Patient	Age (years)	Sex	Disease Duration (years)	Baseline UCDAI Score	Baseline Calprotectin (μg/g)	Disease Extent	Extraintestinal Manifestations	Family Hx	Smoker	Prior Therapy	Ongoing Therapy
1	47	F	26	6	1161	L-sided	iritis/uveitis	None	s/p 13 yrs	5-ASA, Pred, herbal, VSL#3	5-ASA, Chinese herbs, curcumin
2	25	M	6	9	692	Pancolitis	pystomatitis vegetans	None	No	5-ASA, AZA, infliximab	None
3	61	F	40	8	366	Pancolitis	none	Brother and son with UC	1 cig/day	5-ASA, Pred, herbal, VSL#3	5-ASA, boswellia
5	29	M	7	6	280	L-sided	none	Mom with UC	No	5-ASA, VSL#3	5-ASA
6	61	F	22	8	429	L-sided	apthous stomatitis	Mom with UC	No	5-ASA, AZA	None
7	27	F	8	8	209	L-sided	possible sacroiliitis	None	1 cig/week	5-ASA, Entocort, AZA	5-ASA
8	38	F	7	8	ND	Pancolitis	none	None	No	5-ASA, Pred, ABX, AZA, MTX, infliximab, adalimumab	None

5-ASA = 5-aminosalicylic acid preparations, Pred = prednisone, AZA = azathioprine, ABX = antibiotics, MTX = methotrexate, UCDAI = ulcerative colitis disease activity index

**Table 2:** Donor Demographics.

Donor	Age (years)	Sex	Height (cm)	Weight (kg)	BMI (kg/m^2^)	Relationship to Patient	Cohabitation	PMH	Fecal Calprotectin (μg/g)
1	41	M	178	75	23.7	husband	yes	Hypertension	2
2	25	M	191	71	19.5	roommate	yes		26
3	55	M	185	85	24.8	husband	yes		16
3'	55	M	185	85	24.8	father	no		2
6	47	M	183	89	26.6	brother in law	no	Graves' Disease	17
7	28	M	183	88	26.6	ex-boyfriend	no		51
8	37	M	175	69	22.4	husband	yes		ND

BMI = body mass index, PMH = previous medical history

### FMT Safety

Patients were monitored for adverse events throughout the study. Several of the patients reported a mild increase in abdominal cramping and stool output immediately after the transplant. These symptoms had resolved by the follow-up appointment at 1 week. No patient reported fever. The eighth patient presented with persistent abdominal pain 5 days after the procedure. A plain film of the abdomen showed free air suggesting that a micro-perforation had occurred. The patient never developed a fever, tachycardia, or leukocytosis and was managed non-operatively and without the need for antibiotics. The patient was dis-enrolled from the study without further microbiota analysis.

### Clinical, Endoscopic and Histologic Outcomes

**Fig 2:**
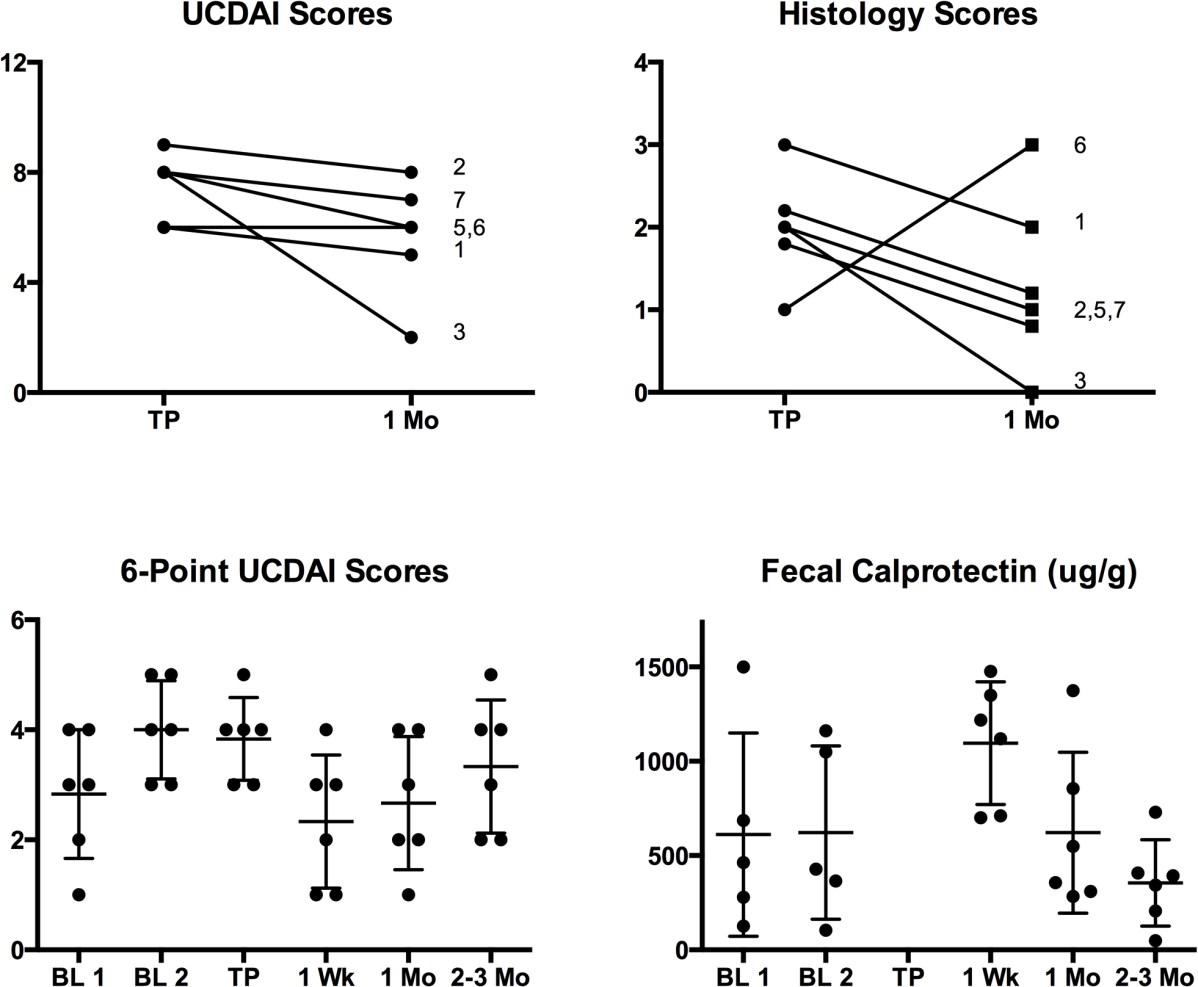
Clinical response to fecal microbiota transplantation. (*A*) Ulcerative colitis disease activity index (UCDAI) scores at transplant (TP) and 1 month (1 Mo) after transplant. (*B*) Histology scores at transplant and 1 month after transplant. (*C*) 6-Point UCDAI Scores (Rectal bleeding + Stool Frequency) at baseline (BL), transplant, and after transplant. (*D*) Fecal calprotectin levels at baseline, transplant, and after transplant. Vertical lines in figures (C) and (D) represent means with standard deviation.

One patient had an improvement in UCDAI score from 8 to 2 and was the only patient to achieve clinical remission at 4 weeks after FMT. (Fig 2A) This patient’s histology score also decreased from 2 to 0 (absence of inflammation). The remaining 5 patients did not achieve clinical response or remission. Six-point UCDAI scores showed a trend toward improvement at 1 week (mean change 1.6) and 4 weeks (mean change 0.8) but the change was not statistically significant. (Fig 2C) All patients eventually had worsening of their symptoms by 3 months, including the patient who had initially achieved remission. Histology scores improved in all but one patient at 4 weeks, but given the small numbers in this study the overall mean change was not statistically significant (Fig 2B). Fecal calprotectin initially increased at 1 week after transplant but then decreased to a level below baseline, although these changes were not statistically significant (Fig 2D).

### Non-Alignment Based Assessment of Donor Similarity and Stability of FMT

**Fig 3:**
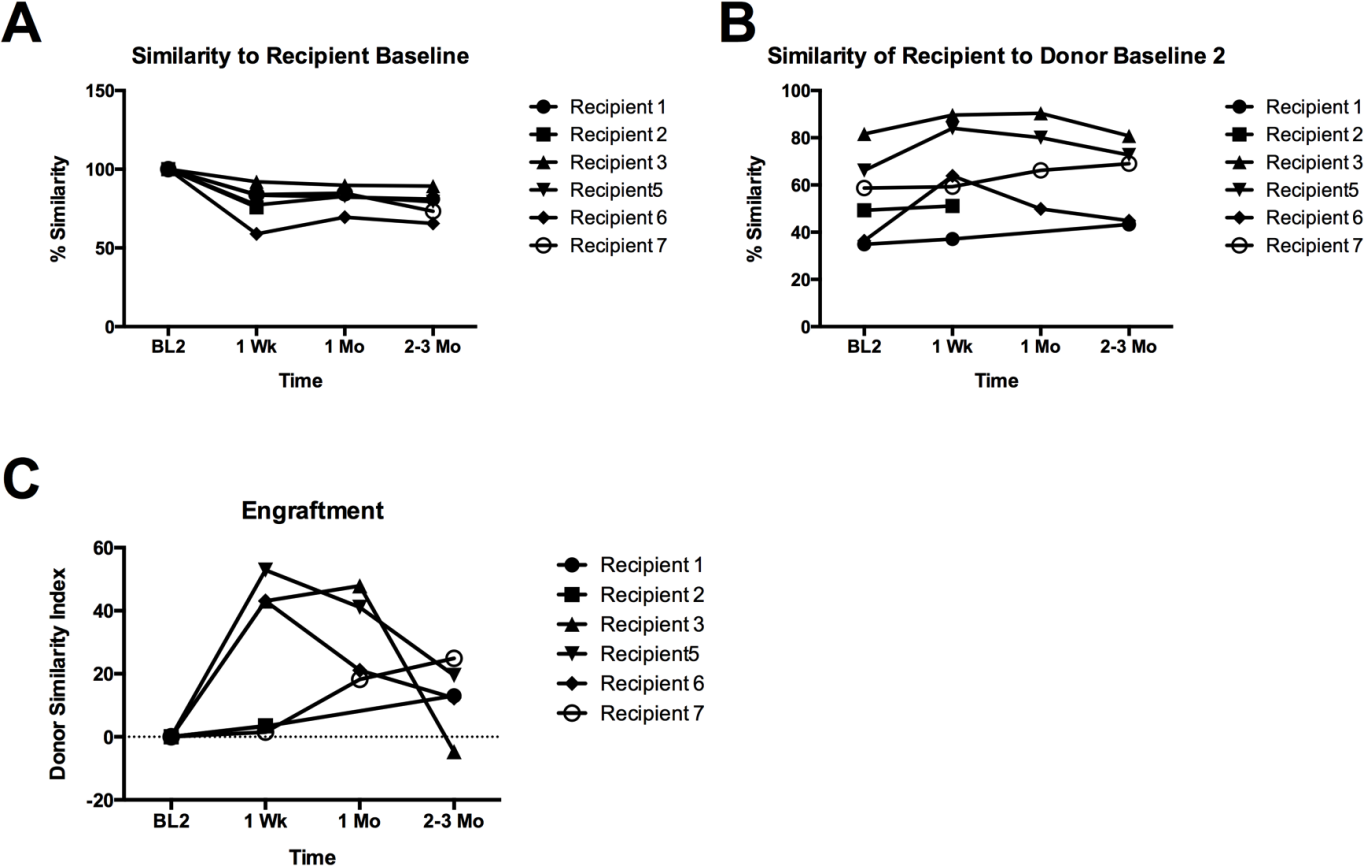
Engraftment and Stability of fecal microbiota transplantation. Recipient similarity to (A) recipient baseline and (B) donor baseline are plotted. The (C) DSI sets the second baseline (BL2) similarity to donor to zero and scales the post-transplant similarities. One hundred percent indicates perfect engraftment.

**Fig 4:**
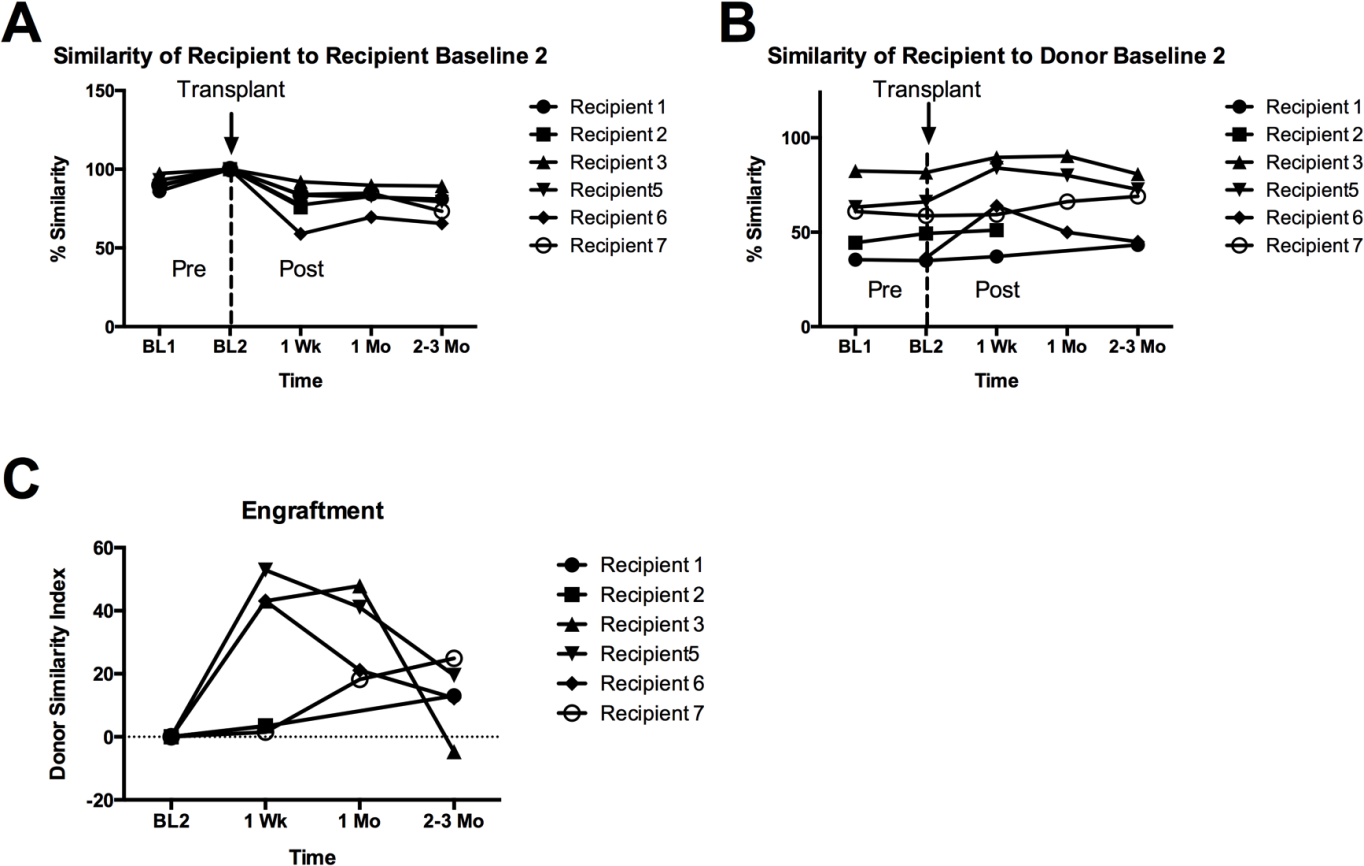
Pairwise comparisons of recipient samples. Pairwise comparison were made between recipient samples before and after transplant (BL 2 and WK 1) to all donor's BL2 samples. In patients that demonstrate transplantation, the percent similarity to their donor (dashed lines) increases more than the percent similarity to any other donor (solid lines). Please note that recipients 3 and 5 had the same donor with stool collected at different time points (D3 and D3’).

We used Compareads, a non-alignment based method to process the metagenomic data and measure DSI of the transplanted microbiota (Fig 3, Table 3). We compared post transplant samples to recipient and donor baseline samples obtained immediately prior to transplant (BL2). We then calculated the DSI for each post transplant time point as described in methods. For some samples, Compareads analysis could not be performed (CNBP) due to high number of human reads and low numbers of bacterial reads. Appropriate controls were used including evaluating baseline change in the microbiota over time prior to transplant. Baseline samples are separated by at least one week and in most cases greater than 1 month. Pairwise comparisons of recipients to all donors were also performed to show that at 1 week, patients that demonstrated donor similarity showed more similarity to their donor than any other donor. (Fig 4)

**Table 3:** Clinical Outcomes Compared with Engraftment Scores.

Subject	Outcomes	BL2	TP	1 Wk	1 Mo	2–3 Mo(s)
R1	Full UCDAI		6		5	
	Histology		3		2	
	6-point UCDAI	5	3	3	2	3
	Engraftment Score			3.4	CNBP	13
R2	Full UCDAI		9		8	
	Histology		2		1	
	6-point UCDAI	3	5	3	4	4
	Engraftment Score			3.5	CNBP	CNBP
R3	Full UCDAI		8		2	
	Histology		2		0	
	6-point UCDAI	4	4	1	1	2
	Engraftment Score			43.1	47.9	-4.7
R5	Full UCDAI		6		6	
	Histology		2		1	
	6-point UCDAI	3	3	2	2	2
	Engraftment Score			52.9	41.1	19.5
R6	Full UCDAI		8		6	
	Histology		1		3	
	6-point UCDAI	4	4	1	5	4
	Engraftment Score			43.1	21.1	12.3
R7	Full UCDAI		8		7	
	Histology		2		1	
	6-point UCDAI	5	4	4	4	5
	Engraftment Score			1.5	18.2	24.9

BL2 = baseline 2, TP = transplant, Wk = week, Mo = month, CNBP = could not be performed

Two patterns of engraftment were observed: moderate increase in DSI with subsequent decline, and low initial increase in DSI with subsequent rise. Recipients 3, 5 and 6 demonstrated the first pattern with moderate increases in DSI’s at 1 week (43%, 53% and 43%) that subsequently decreased by 2–3 months (-4.7, 19.5 and 12.3). It is noteworthy that of the patients demonstrating moderate increase in DSI, only patient 3 showed an increase at 1 month. This is the only patient that went into remission. At 2–3 months she had a flare in her symptoms that correlated with a decrease in her DSI. Recipients 1 and 7 demonstrated the second pattern with low DSI’s at 1 week (3% and 2%) that gradually and only slightly increased by 2–3 months (13% and 25%). No trend could be determined for patient 2 due to the high number of human reads.

### Taxonomic Changes After Transplant

**Fig 5:**
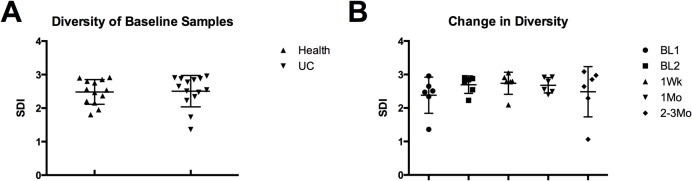
Shannon diversity index (SDI) comparisons of samples. There was no significant difference in baseline recipient and donor diversity. (A) There was also no significant difference in diversity at any time point after transplant relative to baseline. (B) Vertical lines represent means with standard deviation.

We next aimed to determine how the composition of the microbiota changed after transplant. Taxonomic information was mined from the metagenomic data sets using MetaPhlAn and two-hundred and fifteen species were identified across all samples. (S1 Table) A Shannon diversity index was calculated for all samples and no significant difference was found between donor and recipient baselines or between pre- and post-transplant samples. (Fig 5)

Only 19 species demonstrated transplantation in at least one recipient as defined by an increase toward the donor of at least 0.2% from undetectable levels in baseline samples (S1 Table, bolded boxes). Some of these species were only transiently transplanted at 1 week, some persisted at all three time points, and some demonstrated transplantation only in later months. Most of these species belonged to the phyla Actinobacteria and Bacteroidetes. Firmicutes were underrepresented among transplanted species. While there were no significant differences in donor and recipient species after correction for multiple testing, three of the transplanted species did show enrichment (significant P value prior to correction for multiple testing) in healthy donors relative to UC patients including *Alistipes Shahii*, *Gordonibacter pamelaeae*, and *Parabacteroides merdae*, (S2 Table).

The majority of post-transplant increases were in species already present in the recipient at baseline. Changes of one logarithm or greater were common but most of these represented less than 5% in absolute change.(S3 Table, red numbers) There were 22 species that demonstrated a greater than 5% change in absolute abundance. Most notable was an expansion of *Prevotella copri* in patient 2 (2 logs and 75%). There were no trends observed post transplant at higher taxonomic levels.

## Discussion

Our study demonstrates that a single colonoscopic session of FMT without antibiotics did not lead to sustained changes in DSI or clinical improvement in the majority of patients. However, one patient did achieve clinical and histologic remission, suggesting that perhaps a subset of patients may engraft with donor stool and may experience clinical improvement. We made use of a non-alignment based method for comparing two metagenomic sequences. This method avoids any bias associated with mapping reads to taxonomic databases. We show an association of DSI with clinical disease course in the one patient in whom transient clinical remission was achieved. Two other patients who did not achieve remission at 1 month also demonstrated moderate engraftment (DSI>40%) at 1 week; however, unlike the patient that achieved transient remission they showed a decrease in DSI’s at 1 month. While our *a priori* definition of engraftment had been defined as a DSI of greater than 50% at 1 month, we recognize this definition was chosen arbitrarily and post transplant trends in DSI may be more clinically meaningful.

We also performed taxonomic analysis of the metagenomic sequence. After correcting for multiple testing, there were no species that were significantly different between healthy donors and UC recipient. Three species (*Alistipes Shahii*, *Gordonibacter pamelaeae*, and *Parabacteroides merdae*) enriched in our healthy subjects (p value <0.05 prior to correcting for multiple testing) demonstrated transplantation in at least one patient. *Alistipes Shahii and Parabacteroides merdae* belong to the phyla Bacteroidetes and have previously been isolated from appendices of human origin[25] and human stool.[26] *Gordonibacter pamelaeae* belongs to the phyla Actinobacteria and has previously been isolated from the colon of a patient with Crohn’s disease[27] and from the blood of a patient with rectosigmoid carcinoma.[28] While some of these species have paradoxically been isolated from patients with disease, the relevance of these transplanted species to disease course is unclear and further evaluation of specific strains in patients from our study in models of inflammation could be useful.

Clinically, our results are similar to other small prospective studies of FMT for UC in which response to therapy is minimal and transient.[29–31] These results are different from studies of FMT for *C*. *difficile* colitis in which a single FMT session leads to sustained cure rates of greater than 90%.[10] They are also contrary to engraftment studies in CDI and animal models showing that transplanted microbiota are stable for up to a year.[32,33] The lack of sustained response in most patients with UC is consistent with an underlying pathophysiology different from that of *C*. *difficile*. Rather than being driven by a single pathogen, UC is thought to result from a complex interaction of dysbiotic microbiota with an aberrant immune system. The above results suggest that a therapeutic FMT approach to UC will be different from that for *C*. *difficile*.

Further studies are warranted to identify the subset of patients with UC who are most likely to respond to FMT, and to determine the optimal FMT administration protocol. Pre-transplant bowel lavage and antibiotics, fecal processing method (blended vs. mixed), fecal transmission route (nasoenteric vs. colonoscopic vs. enema), treatment length (single administration vs. serial FMT), and donor selection might all play a role in augmenting FMT engraftment that could allow greater efficacy.

Antibiotics have the potential to remove entrenched dysbiotic species in a process akin to conditioning chemotherapy. Antibiotics would ideally be highly selective as they come with the risk of further depleting protective commensals and opening up microbial niches for further dysbiosis. A study by Angelberger *et al*.[30] in which metronidazole was administered did not have improved outcomes, but these results must be interpreted in the context of the more severely affected patient population evaluated (moderate to severe) and the fact that patients had their baseline UC therapy stopped prior to FMT. It is also possible that metronidazole lacked the appropriate spectrum of antimicrobial activity.

An alternative to antibiotics is a more robust inoculum of healthy commensals in the form of serial FMT and maintenance therapy. This might serve to not only populate the recipient with health promoting bacteria, but also more effectively deplete entrenched inflammatory species via niche exclusion or bacteriocin production in a process akin to graft versus host effects. Serial transplantation in UC has been championed by some FMT experts[34] and in a limited form was undertaken by Kunde *et al*. in a study evaluating serial enemas for 5 days in children. [29] This paper had one of the greatest clinical responses to FMT of published prospective studies, but these results must be interpreted in the context of the mild to moderately affected pediatric population that was evaluated.

In regard to donor selection, we did not identify any donor criteria that trended with successful transplantation, although the study was not powered to detect such associations. Specifically, we did not find a correlation between baseline microbiota similarity, fecal calprotectin, age, relationship to donor, or cohabitation. It is noteworthy that both recipient 3 and 5 had the same donor and both achieved robust DSI increases.

Regarding adverse events, one patient developed a micro-perforation after FMT. This was managed non-operatively and without need for hospitalization or antibiotics, as she remained clinically stable and was improving at the time of presentation several days after the procedure. Perforation is a known complication of colonoscopy. It is unlikely that administration of exogenous stool caused the micro-perforation although it is possible that FMT may have contributed to a greater inflammatory response in an otherwise subclinical event. As in other studies, our patients experienced self-limited mild epigastric discomfort and increased stool frequency immediately following the FMT procedure. Our patients also showed a paradoxical increase in fecal calprotectin at 1 week that normalized by 1 month. This increase was not statistically significant.

There are many limitations to any clinical interpretations of our study. It was designed to evaluate engraftment and not as an efficacy trial. As such, it is uncontrolled and includes a small number of patients limited to mildly to moderately active disease. As a single center study, we cannot exclude investigator bias although we took measures to make endpoints as objective as possible including fecal calprotectin measurement and blinded histology scores. While no antibiotics were used in this study prior to FMT, we cannot exclude that the PEG bowel preparation influenced histology scores and microbiota profiles. We did not control for diet or fecal transit time and cannot rule out their impact on the intestinal microbiome and disease activity. With these limitations in mind, our study suggests that there may be a limited response to FMT in a subset of patients with UC and that improvement in engraftment could improve efficacy. Further investigation is warranted to determine the optimal patient and donor selection and FMT administration protocol for UC.

## Supporting Information

S1 TREND Statement Checklist Trend Statement Checklist. (PDF)

S1 Protocol Study Protocol. (PDF)

S1 Questionnaire Full Length Donor History Questionnaire. (PDF)

S1 Table Heat Map of Relative Species Abundance Acros all Samples. (PDF)

S2 Table Differences in Percent Abundance of Species at Baseline Between Donor and Recipient. (PDF)

S3 Table Relative and Absolute Changes in Species. (PDF)
