# Supplementary material for: Low Level Engraftment and Improvement following a Single Colonoscopic Administration of Fecal Microbiota to Patients with Ulcerative Colitis
Source: PLoS One. 2015 Aug 19;10(8):e0133925. doi: 10.1371/journal.pone.0133925 (PMC4544847; doi:10.1371/journal.pone.0133925)
Supplement: S1 Protocol — (PDF) [file pone.0133925.s002.pdf]

Evaluation of durability of Fecal Microbiota Transplantation in Patients with Mild to Moderate Ulcerative Colitis

Version Date:	10.11.12
IRB #:	HSD #41454
Investigational Product:	Fecal Microbiota Transplantation
IND Number:	N/A
Development Phase:	Phase 1
Sponsor:	Tim Zisman, MD<br>1959 NE Pacific Street, Box 356424<br>Seattle, WA 98103
Funding Organization:	Broad Institute
Principal Investigator:	Name: Tim Zisman<br>Telephone: (206) 598-4377<br>Fax: (206) 685-8684<br>E-mail: tzisman@medicine.washington.edu
Research Manager:	Name: Kelly Crowder<br>Telephone: (206) 543-3220<br>Fax: (206) 221-1356<br>E-mail: kcrowder@medicine.washington.edu

Approval:

PI or Sponsor Signature (Name and Title) *Date*

This confidential information about an investigational product is provided for the exclusive use of investigators of this product and is subject to recall at any time. The information in this document may not be disclosed unless federal or state law or regulations require such disclosure. Subject to the foregoing, this information may be disclosed only to those persons involved in the study who have a need to know, with the obligation not to further disseminate this information.

TABLE OF CONTENTS

1 BACKGROUND 4
2 STUDY RATIONALE 6
2.1 Risk / Benefit Assessment 6
3 STUDY OBJECTIVES 7
3.1 Primary Objective 7
3.2 Secondary Objectives 7
4 STUDY DESIGN 7
4.1 Study Overview 7
5 CRITERIA FOR EVALUATION 8
5.1 Primary Efficacy Endpoint 8
5.2 Secondary Efficacy Endpoints 8
5.3 Safety Evaluations 8
6 SUBJECT SELECTION 8
6.1 Study Population 8
6.2 Inclusion Criteria 8
6.3 Exclusion Criteria 8
7 CONCURRENT MEDICATIONS 9
7.2 Prohibited 10
8 STUDY TREATMENTS 10
9 STUDY PROCEDURES AND GUIDELINES 10
9.1 Clinical Assessments 10
9.2 Clinical Laboratory Measurements 11
9.4 Research Laboratory Measurements 11
10 EVALUATIONS BY VISIT 11
11 ADVERSE EXPERIENCE REPORTING AND DOCUMENTATION 14
11.5 Safety Management Plan 4
12 DISCONTINUATION AND REPLACEMENT OF SUBJECTS 14
12.3 Replacement of Subjects 14
13 PROTOCOL VIOLATIONS 14
17 ADMINISTRATIVE, ETHICAL, REGULATORY CONSIDERATIONS 14
17.1 Protocol Amendments 14
17.2 Institutional Review Boards and Independent Ethics Committees 15
17.3 Informed Consent Form 15
17.4 Publications 16
17.5 Investigator Responsibilities 16

PROTOCOL SYNOPSIS

**TITLE**	Evaluation of Durability of Fecal Microbiota Transplantation in Patients with Mild to Moderate Ulcerative Colitis
**SPONSOR**	Tim Zisman, MD
**FUNDING ORGANIZATIONS**	Broad Institute; University of Washington Division of Gastroenterology Clinical Translational Research Core
**NUMBER OF SITES**	1
**RATIONALE**	Fecal microbiota therapy (FMT) is an emerging treatment for gastrointestinal disorders marked by an imbalance in the intestinal microbial flora (dysbiosis). It is hypothesized to work by shifting the recipient's microbiota toward a eubiotic microbial community that resists colonization by pathogenic organisms or decreases its inherent inflammatory properties. Several studies now report its efficacy in treatment of severe *Clostridium difficile* colitis. Preliminary studies using FMT in Ulcerative Colitis (UC) have also met with some success. This is corroborated by several lines of evidence suggesting dysbiosis plays an important role in UC pathogenesis. While a recent study using FMT in patients with irritable bowel syndrome (IBS) and constipation found transplants persist for up to 2 years, the extent to which the microbiota is alterable in UC is not known. Indeed, there may be particular genetic or immunologic factors in UC leading to selection pressure preventing a change in the microbiota. As an initial step into investigating the potential efficacy of stool transplants for Ulcerative Colitis (UC), we propose to determine the feasibility and stability of transplanted microbiota in a series of 10 patients with mild to moderate UC.
**STUDY DESIGN**	This is a prospective cohort study.
**PRIMARY OBJECTIVE**	1. Determine the feasibility and durability of fecal microbiota therapy in patients with mild to moderate UC.
**SECONDARY OBJECTIVES**	1. Determine what donor and recipient factors correlate with engraftment.<br>2. Determine if there is any endoscopic or clinical improvement in UC disease activity.
**NUMBER OF SUBJECTS**	10 Recipients and 10 donors.
**RECIPIENT SELECTION CRITERIA**	<u>Inclusion Criteria</u>:<br>1. Mild to moderate UC.

	Exclusion Criteria:<br>1. Antibiotic exposure in the last 3 months.<br>2. Biologic or immunomodulatory therapy within the last 3 months.<br>3. Corticosteroid therapy or probiotics within the last 2 weeks.<br>4. Severely active disease (defined as UCDAI scores of 10 or greater, or patients with endoscopic disease activity scores of 3 or greater).
**DONOR SELECTION CRITERIA**	Inclusion Criteria:<br>1. Intimate contact, family member, or friend of recipient.<br><br>Exclusion Criteria by Questionnaire:<br>1. Known human immunodeficiency virus (HIV), hepatitis B or C infections<br>2. Known exposure to HIV or viral hepatitis (within the previous 12 months)<br>3. High-risk sexual behaviors (examples: sexual contact with anyone with HIV/acquired immune deficiency syndrome or hepatitis, men who have sex with men, sex for drugs or money)<br>4. Use of illicit drugs<br>5. Tattoo or body piercing within 6 months<br>6. Incarceration or history of incarceration<br>7. Known current communicable disease (eg, upper respiratory tract infection)<br>8. Risk factors for variant Creutzfeldt Jakob disease<br>9. Travel (within the last 6 months) to areas of the world where diarrheal illnesses are endemic or risk of traveler's diarrhea is high<br>10. History of inflammatory bowel disease<br>11. History of IBS, idiopathic chronic constipation, or chronic diarrhea<br>12. History of gastrointestinal malignancy or known polyposis<br>13. Antibiotics within the preceding 3 months<br>14. Major immunosuppressive medications, eg, calcineurin inhibitors, exogenous glucocorticoids, biological agents, etc (unless patient has had a washout period)<br>15. Systemic antineoplastic agents<br>16. Recent ingestion of a potential allergen (eg, nuts) where recipient has a known allergy to this (these) agent(s).<br>17. Positive results on any of the following screening tests: HIV, HAV, HBV, HCV, C.diff PCR, stool enterics, fecal giardia antigen, cryptosporidium, cyclosporo, isospora, ova and parasites, and syphilis panel.
**TEST PRODUCT, DOSE, AND ROUTE OF ADMINISTRATION**	300cc of stool mixture (3ml:1gm of normal saline:stool) delivered to terminal ileum and cecum by colonoscope.

**CONTROL PRODUCT, DOSE AND ROUTE OF ADMINISTRATION**	No Control.
**DURATION OF SUBJECT PARTICIPATION AND DURATION OF STUDY**	Subjects will be on study for up to 4 months<br>**Screening:** 2 to 4 weeks<br>**Treatment:** 1 day<br>**Follow-up:** 3 months<br>The total duration of the study is expected to be 12 months. 8 months for subject recruitment and 4 months for final subject follow-up.
**CONCOMMITANT MEDICATIONS**	Prohibited: Probiotics (2 weeks), Corticosteroids (2 weeks), Biologics (3 months), Immuno-modulators (3 months), Antibiotics (3months)<br>Allowed: Aminosalicylates
**EFFICACY EVALUATIONS**	Microbiota Engraftment: 16S rRNA sequencing, fluorescence *in situ* hybridization, and culture of stool and biopsies.<br>Clinical improvement: UCDAI Scores, histology of biopsies, and fecal calprotectin
PRIMARY ENDPOINT	• Engraftment of microbiota
SECONDARY ENDPOINTS	• Clinical improvement in UC
**SAFETY EVALUATIONS**	Patient will be instructed to call the DDC with any change from baseline including worsened abdominal pain, fever, chills, worsened diarrhea. CBC, CMP, ESR, and CRP as well as physical exam and patient history will be performed at 3 months.
**STATISTICS**<br>**Primary Analysis Plan**	Both weighted and unweighted UniFrac distances will be calculated in this study. A relative difference of UniFrac distances comparing recipient and donor stool at baseline (inter-individual) and comparing recipient stool prior to transplant at two time points (intra-individual) for each donor recipient pair will be calculated. This value multiplied by 50% will be the threshold value (TV) to determine if the transplant engrafts and is stable. UniFrac distances comparing the recipient pre- and post- transplant will need to be >(intra-individual variation of the recipient+TV). In addition, UniFrac distances comparing the recipient post-transplant with the donor will need to be <(intra-individual variation of the donor+TV). Engraftment will be defined as meeting both of these criteria at 7 days. Stability will be defined as meeting both these criteria at 1 month or 3 months.

1 BACKGROUND

FMT is an emerging therapy that has been gaining considerable recent attention (Khoruts A 2011). It has been shown through several trials to be an effective treatment for refractory *Clostridium difficile* colitis (Surawicz 2011). Analysis of the microbiota in a patient with *C. difficile* post FMT demonstrated a change in the microbiota that was more reflective of the donor than the recipient (Khoruts et al 2010). In a series of 10 patients undergoing FMT with a spectrum of GI diseases including IBS, constipation, and Crohn's disease, the post transplant microbiota was found to be more similar to the donor stool as long as 2 years after transplant. (Grehan MJ 2010)

There are reports of FMT being used successfully to treat refractory ulcerative colitis. (Borody et al 2003). Dr. Borody reports on 6 patients in whom FMT was curative of severe refractory ulcerative colitis for up to 13 years off conventional treatment. Based on personal communication, these six patients were subset of 40 patients. Interestingly, in many of the remaining patients, serial transplantation was required to achieve clinical remission.

2 STUDY RATIONALE

Dysbiosis of endogenous gastrointestinal microbiota may play an important role in the pathogenesis of UC (Sartor et al 2008). Molecular studies of the microbiota using 16S rRNA technology have revealed an overall decrease in biodiversity as well as specific shifts in bacterial populations, including a decrease in Firmicutes (especially Clostridial species), and an increase in Proteobacteria (especially E.coli) (Peterson et al 2008). Mouse models for UC with genetically engineered defects in their immune system treated with antibiotics or raised without gut bacteria have shown that the gut microbiota is essential for eliciting the UC phenotype. (Kang et al. 2008). Administration of various probiotic formulations to patients have been successful in inducing remission in mild-to-moderate UC. (Sood et al 2009, Tursi et al 2010).

While transplants in other GI disorders have persisted for up to two years, the extent to which the microbiota in UC is alterable and durable is not known. Indeed, the patient's microbiota may, in part, be under genetically or immunologically determined selection pressure of the patient's immune system and may resist change. In this study, we aim to determine the engraftment durability in 10 patients as an initial step to investigating FMT's therapeutic potential for UC.

2.1 Risk / Benefit Assessment

FMT for other indications including *C.difficile* is well tolerated. There are no adverse events reported in the literature to date. The risks associated with this study include the following:

1. Risk of contracting an infectious agent from the stool transplant. We will be screening the donor with American Association of Blood Banks Questionnaire and

with the below mentioned stool and serology tests to limit the possibility of transmission of infection.

2. Risk of bleeding, perforation, infection, side effects from sedating medications from the colonoscopy and sigmoidoscopy with biopsy. The colonoscopy is standard of care. The flexible sigmoidoscopy with biopsy is part of the study protocol and does increase risk of bleeding perforation, and infection. This procedure will be performed unsedated so the added risk of sedation does not pertain.

3. Risk of progressive disease. Patients who are already on 5-ASA will be maintained on 5-ASA. There may be some patients in whom we discontinue biologics or immunosuppressive medications prior to enrollment and there is the risk of worsened disease. There is also the risk of worsened disease in not progressing with standard escalation therapy during the duration of the study.

3 STUDY OBJECTIVES

3.1 Primary Objective

1. The primary objective is to determine the feasibility and durability of fecal microbiota therapy in patients with mild to moderate UC.

3.2 Secondary Objectives

1. Determine if there is any clinical improvement in UC as measured by endoscopic appearance, histology, UCDAI scores, and fecal calprotectin levels.
2. Determine what donor and recipient factors correlate with engraftment.

4 STUDY DESIGN

4.1 Study Overview

This is a single center, prospective cohort study. 10 recipient/donor pairs are planned. Each recipient will undergo a single session of FMT. Biopsies will be collected immediately prior to FMT and 1 month afterward. Stool will be collected at 2 time points (1 week apart) before FMT and 3 time points after FMT (1week, 1 month, 3 months). Donor stool will be collected at two time points before FMT.

Screening data will be reviewed to determine subject eligibility. Subjects who meet all inclusion criteria and none of the exclusion criteria will be entered into the study.

The following treatment regimen will be used:

- 300cc of stool mixture (3ml:1grams of normal saline: donor stool)

Total duration of subject participation will be 4 months. Total duration of the study is expected to be 1 year.

5 CRITERIA FOR EVALUATION

5.1 Primary Endpoint

- Engraftment of donor microbiota as measured by UniFrac distances from baseline to 1 month.

5.2 Secondary Endpoints

- Engraftment of donor microbiota as measured by UniFrac distances from baseline to 3 months.
- Clinical improvement in UC disease activity as measured by UCDAI.
- Decrease in fecal calprotectin level

5.3 Safety Evaluations

- Patients will be assessed at a 3 month evaluation with a physical exam and labs including CBC, CMP, ESR, and CRP. We do not expect adverse events given there are no adverse events reported in the literature of FMT.

6 SUBJECT SELECTION

6.1 Study Population

Subjects with a diagnosis of ulcerative colitis who meet the inclusion and exclusion criteria will be eligible for participation in this study. Donors will be a significant other, family member or friend of the recipient and will also need to meet exclusion and inclusion criteria.

6.2 Recipient Inclusion Criteria

1. Male or female ≥ 18 years of age at Visit 1.
2. Prior documentation of a UC diagnosis as evidenced by endoscopic and histologic evaluation.
3. Written informed consent obtained from subject and ability for subject to comply with the requirements of the study.

6.3 Recipient Exclusion Criteria

1. Presence of a condition or abnormality that in the opinion of the Investigator would compromise the safety of the patient or the quality of the data.
2. Antibiotic exposure in the last 3 months.
3. Biologic or immunomodulatory therapy within the last 3 months.
4. Corticosteroid therapy or probiotic usage within the last 2 weeks.
5. Severely active disease (defined as UCDAI scores of 10 or greater, or patients with endoscopic disease activity scores of 3 or greater).

6.4 Donor Inclusion Criteria

1. Significant other, family member or friend of recipient.
2. Written informed consent obtained from subject and ability for subject to comply with the requirements of the study.

6.5 Donor Exclusion Criteria

1. Presence of a condition or abnormality that in the opinion of the Investigator would compromise the safety of the patient or the quality of the data.
2. Known human immunodeficiency virus (HIV), hepatitis B or C infections
3. Known exposure to HIV or viral hepatitis (within the previous 12 months)
4. High-risk sexual behaviors (examples: sexual contact with anyone with HIV/acquired immune deficiency syndrome or hepatitis, men who have sex with men, sex for drugs or money)
5. Use of illicit drugs
6. Tattoo or body piercing within 6 months
7. Incarceration or history of incarceration
8. Known current communicable disease (eg, upper respiratory tract infection)
9. Risk factors for variant Creutzfeldt Jakob disease
10. Travel (within the last 6 months) to areas of the world where diarrheal illnesses are endemic or risk of traveler's diarrhea is high
11. History of inflammatory bowel disease
12. History of IBS, idiopathic chronic constipation, or chronic diarrhea
13. History of gastrointestinal malignancy or known polyposis
14. Antibiotics within the preceding 3 months
15. Major immunosuppressive medications, eg, calcineurin inhibitors, exogenous glucocorticoids, biological agents, etc (unless patient has had a washout period)
16. Systemic antineoplastic agents
17. Recent ingestion of a potential allergen (eg, nuts) where recipient has a known allergy to this (these) agent(s).
18. Positive results on any of the following screening tests: HIV, HAV, HBV, HCV, C.diff PCR, stool enterics, fecal giardia antigen, cryptosporidium, cyclospora, isospora, ova and parasites, and syphilis panel.

7 CONCURRENT MEDICATIONS

All subjects should be maintained on the same medications throughout the entire study period, as medically feasible, with no introduction of new chronic therapies.

7.1 Medications and Treatments

Standard therapy for UC is allowed except for treatments noted in the exclusion criteria described above and as noted in the prohibited medications section below.

The following medications are prohibited during the study and administration will be considered a protocol violation.

- Steroids (2 week washout)
- Probiotics (2 week washout)
- Immunomodulators (3 month washout)
- Biologics (3 month washout)
- Antibiotics (3 month washout)

The following medications are allowed during the study.

- Aminosalicylates

8 STUDY TREATMENTS

Study will involve administration of 300cc of a stool saline mixture. The stool saline mixture will be prepared by adding 3ml of sterile normal saline to 1mg of donor stool. The full 300cc of stool saline mixture will be strained through two 4x4 gauze sheets to remove particulate matter that could clog the colonoscope. Five 60cc syringes will be loaded with the mixture and administered to the terminal ileum and right colon. After colonoscopy that patient will be administered loperamide 4mg PO times one and will lye recumbent for 3 hours.

9 STUDY PROCEDURES AND GUIDELINES

A Schedule of Events representing the required testing procedures to be performed for the duration of the study is diagrammed in Appendix 1 and Appendix 2.

Prior to conducting any study-related activities, written informed consent and the Health Insurance Portability and Accountability Act (HIPAA) authorization must be signed and dated by the donor and recipient.

9.1 Clinical Assessments

9.1.1 Concomitant Medications

All concomitant medication and concurrent therapies will be documented at baseline and at the 3 month visit. Dose, route, unit frequency of administration, and indication for administration and dates of medication will be captured.

9.1.2 Demographics

Demographic information (date of birth, gender, race) will be recorded at baseline.

9.1.3 Medical History

Relevant medical history, including history of current disease, and information regarding underlying diseases will be recorded at baseline.

9.1.4 Physical Examination

A complete physical examination will be performed by either the investigator or a subinvestigator who is a physician at baseline. Qualified staff (MD, NP, RN, and PA) may complete the abbreviated physical exam at all other visits. New abnormal physical exam findings must be documented and will be followed by a physician or other qualified staff at the next scheduled visit.

9.1.5 Vital Signs

Body temperature, blood pressure, pulse and respirations will be performed after resting for 5 minutes at baseline and the 3 month visit.

9.1.6 Adverse Events

Information regarding occurrence of adverse events will be captured throughout the study. Duration (start and stop dates), severity/grade, outcome, treatment and relation to FMT will be recorded.

9.2 Clinical Laboratory Measurements

9.2.1 Blood

Recipient: Blood will be obtained and sent to the clinical lab for a complete blood count (CBC)(hemoglobin, hematocrit, red blood cell count, white blood cell count, white blood cell differential, and platelet count), comprehensive metabolic panel (CMP)(sodium, potassium carbon dioxide, chloride, blood urea nitrogen, creatinine, serum glucose, calcium, total protein, albumin, alkaline phosphatase, alanine amino transferase, aspartate amino transferase, bilirubin), erythrocyte sedimentation rate (ESR), and serum C-reactive protein (CRP).

9.2.2 Stool Tests

Recipient: Stool will be obtained and sent for *C.difficile* PCR (CDTP). If patient has a recent travel history stool will also be sent for routine culture and O&P.

9.3 Research Laboratory Measurements (include sections as appropriate)

9.3.1 Blood

Donor: Blood will be obtained and sent to the clinical lab for HIV antigen and antibody (HVAGAB), hepatitis A, B, and C panel (HABC), Hepatitis B core IgM Antibody (HBCM). Non-intimate donor's blood will also be sent for serological syphilis panel (SYPHS).

9.3.2 Stool Tests

Recipient: Stool will be obtained and sent for fecal calprotectin.

Donor: All donor's stool will be sent for *C.difficile* PCR (CDTP). Non-intimate donor stool will also be sent for routine bacterial culture plus *E.coli* 0157 (STOCEC), Giardia antigen (SGRDAG), cryptosporidium/cyclospora, isospora (CYCLOP), and Ova & Parasites (O&P).

10 EVALUATIONS BY VISIT

10.1 Recipient Visit (Week -4)

1. Review the study with the subject and obtain written informed consent and HIPAA authorization.
2. Assign the recipient a unique study number.
3. Record demographics data.
4. Record medical history, including a history of ulcerative colitis, diagnosis date, and prior treatments.
5. Patient to be asked UCDAI questions.
6. Record concomitant medications.
7. Perform a complete physical examination.
8. Perform and record vital signs.
9. Collect blood for clinical laboratory tests. (CBC, CMP, CRP, ESR)
10. Send patient home with stool collection kit.
11. Send patient home with donor consent and ask to identify a donor.

10.2 Donor Visit 1 (Week -2)

1. Donor screened by phone questionnaire prior to visit.
2. Review the study with the subject and obtain written informed consent and HIPAA authorization.
3. Assign the donor a unique study number.
4. Collect blood for clinical laboratory tests. (HVAGAB, HABC, and HCBM for all donors. SYPHS for non-intimate donors)
5. Schedule recipient appointments for colonoscopy (date = D),
clinic visit (D + 7 days), flex sig (D +28 days), and clinic visit (D + 12 weeks)
6. Send donor home with first stool collection kit.

10.3 Recipient Visit 1 (Week -2)

1. Patient to drop off first stool sample for C.diff. PCR, fecal calprotectin, and research processing. (RNAlater, straw into modified Carnoy solution, and fresh stool)
2. Send patient home with second stool collection kit.

10.4 Donor Visit 2 (Week -1)

1. Donor to drop off first stool sample for fecal calprotectin, CDTP, and research processing on all subjects. Non-intimate contacts should also be sent for (STOCEC, SGRDAG, CYClOP, and OAPP).

2. Send donor home with second stool collection kit and 60cc of Milk of Magnesia to be taken the night before the transplant.

10.5 Recipient Visit 2 (Week -1)

1. Patient to drop off second stool sample for research processing.
2. Patient to be asked UCDAI Questions

10.6 Donor & Recipient Visit 3 (Week 0)

1. Donor to drop off second stool sample for transplant and research processing.
2. Patient to be asked UCDAI Questions
3. Perform a limited physical examination.
4. Perform and record vital signs.
5. Recipient to have colonoscopy with biopsy.
6. Stool transplant.
7. Send recipient home with third stool collection kit.

10.7 Recipient Visit 4 (Week 1)

1. Patient to drop off third stool sample for fecal calprotectin and research processing.
2. Patient to be asked UCDAI questions.
3. Record concomitant medications.
4. Perform a complete physical examination.
5. Perform and record vital signs.
6. Send patient home with fourth stool collection kit.

10.8 Recipient Visit 5 (Week 4)

1. Patient to drop off fourth stool sample for fecal calprotectin and research processing.
2. Patient to be asked UCDAI questions.
3. Flexible sigmoidoscopy with biopsies
4. Send patient home with fifth stool collection kit.

10.9 Recipient Visit 6 (Week 12)

1. Patient to drop off fifth stool sample for fecal calprotectin and research processing.
2. Patient to be asked UCDAI questions.
3. Record concomitant medications.
4. Perform a complete physical examination.
5. Perform and record vital signs.
6. Collect blood for clinical laboratory tests. (CBC, CMP, CRP, ESR)

11 ADVERSE EXPERIENCE REPORTING AND DOCUMENTATION

Dr. Tim Zisman should be contacted directly at this number to report medical concerns or questions regarding safety.

Phone: (206) 598-4377

12 DISCONTINUATION AND REPLACEMENT OF SUBJECTS

12.1 Withdrawal of Subjects from the Study

A subject may be withdrawn from the study at any time if the subject, the investigator, or the Sponsor feels that it is not in the subject's best interest to continue.

All subjects are free to withdraw from participation at any time, for any reason, specified or unspecified, and without prejudice.

Reasonable attempts will be made by the investigator to provide a reason for subject withdrawals. The reason for the subject's withdrawal from the study will be specified in the subject's source documents.

12.2 Replacement of Subjects

Subjects who withdraw from the study will be replaced.

13 PROTOCOL VIOLATIONS

A protocol violation occurs when the subject fails to adhere to significant protocol requirements affecting the inclusion, exclusion, subject safety and primary endpoint criteria. Protocol violations for this study include, but are not limited to, the following:

- Failure to meet inclusion/exclusion criteria
- Use of a prohibited concomitant medication

14 ADMINISTRATIVE, ETHICAL, REGULATORY CONSIDERATIONS

The study will be conducted according to the Declaration of Helsinki, Protection of Human Volunteers (21 CFR 50), Institutional Review Boards (21 CFR 56), and Obligations of Clinical Investigators (21 CFR 312).

To maintain confidentiality, all laboratory specimens, evaluation forms, reports and other records will be identified by a coded number and initials only. All study records will be kept in a locked file cabinet and code sheets linking a patient's name to a patient identification number will be stored separately. Clinical information will not be released without written permission of the subject, except as necessary for monitoring by the FDA. The Investigator must also comply with all applicable privacy regulations (e.g., Health Insurance Portability and Accountability Act of 1996).

14.1 Protocol Amendments

Protocol amendments cannot be implemented without prior written IRB/IEC approval except as necessary to eliminate immediate safety hazards to patients. A protocol

amendment intended to eliminate an apparent immediate hazard to patients may be implemented immediately, provided the IRBs are notified within five working days.

14.2 Institutional Review Boards and Independent Ethics Committees

The protocol and consent form will be reviewed and approved by the IRB/IEC of each participating center prior to study initiation. Serious adverse experiences regardless of causality will be reported to the IRB/IEC in accordance with the standard operating procedures and policies of the IRB/IEC, and the Investigator will keep the IRB/IEC informed as to the progress of the study. The Investigator will obtain assurance of IRB/IEC compliance with regulations.

Protocol and/or informed consent modifications or changes may not be initiated without prior written IRB/IEC approval except when necessary to eliminate immediate hazards to the patients or when the change(s) involves only logistical or administrative aspects of the study. Such modifications will be submitted to the IRB/IEC and written verification that the modification was submitted and subsequently approved should be obtained.

The IRB/IEC must be informed of revisions to other documents originally submitted for review; serious and/or unexpected adverse experiences occurring during the study in accordance with the standard operating procedures and policies of the IRB; new information that may affect adversely the safety of the patients of the conduct of the study; an annual update and/or request for re-approval; and when the study has been completed.

14.3 Informed Consent Form

Informed consent will be obtained in accordance with the Declaration of Helsinki, ICH GCP, US Code of Federal Regulations for Protection of Human Subjects (21 CFR 50.25[a,b], CFR 50.27, and CFR Part 56, Subpart A), the Health Insurance Portability and Accountability Act (HIPAA, if applicable), and local regulations.

The Investigator will prepare the informed consent form and HIPAA authorization and provide the documents to the Sponsor or designee for approval prior to submission to the IRB/IEC. The consent form generated by the Investigator must be acceptable to the Sponsor and be approved by the IRB/IEC. The written consent document will embody the elements of informed consent as described in the International Conference on Harmonisation and will also comply with local regulations. The Investigator will send an IRB/IEC-approved copy of the Informed Consent Form to the Sponsor (or designee) for the study file.

A properly executed, written, informed consent will be obtained from each subject prior to entering the subject into the trial. Information should be given in both oral and written form and subjects must be given ample opportunity to inquire about details of the study. If appropriate and required by the local IRB/IEC, assent from the subject will also be obtainedA copy of the signed consent form will be given to the subject and the original will be maintained with the subject’s study records.

14.4 Publications

The publication or presentation of any study results shall comply with all applicable privacy laws, including, but not limited to, the Health Insurance Portability and Accountability Act of 1996.

14.5 Investigator Responsibilities

By signing the Agreement of Investigator form, the Investigator agrees to:

1. Conduct the study in accordance with the protocol and only make changes after notifying the Sponsor (or designee), except when to protect the safety, rights or welfare of subjects.
2. Personally conduct or supervise the study (or investigation).
3. Ensure that the requirements relating to obtaining informed consent and IRB review and approval meet federal guidelines, as stated in § 21 CFR, parts 50 and 56.
4. Report to the Sponsor or designee any AEs that occur in the course of the study, in accordance with §21 CFR 312.64.
5. Ensure that all associates, colleagues and employees assisting in the conduct of the study are informed about their obligations in meeting the above commitments.
6. Maintain adequate and accurate records in accordance with §21 CFR 312.62 and to make those records available for inspection with the Sponsor (or designee).
7. Ensure that an IRB that complies with the requirements of §21 CFR part 56 will be responsible for initial and continuing review and approval of the clinical study.
8. Promptly report to the IRB and the Sponsor (or designee) all changes in the research activity and all unanticipated problems involving risks to subjects or others (to include amendments and IND safety reports).
9. Seek IRB approval before any changes are made in the research study, except when necessary to eliminate hazards to the patients/subjects.
10. Comply with all other requirements regarding the obligations of clinical investigators and all other pertinent requirements listed in § 21 CFR part 312.

APPENDIX 1. STUDY FLOW SHEET

	Week -4	*Week -2*	*Week -1*	*Week 0*	*Week 1*	*Week 4*	*Week 12*
**Recipient**	Consent<br>Blood<br>UCDAI<br>Clinic Visit	R. Stool 1<br>UCDAI	R. Stool 2<br>UCDAI	UCDAI<br>Col. w/ Bx	R. Stool 3<br>UCDAI<br>Clinic Visit	R. Stool 4<br>UCDAI<br>Sig.w/ Bx	R. Stool 5<br>Blood<br>UCDAI<br>Clinic Visit
**Donor**	Ph.Screen	Consent<br>Blood	D. Stool 1	D. Stool 2			
