# Supplementary material for: Low Level Engraftment and Improvement following a Single Colonoscopic Administration of Fecal Microbiota to Patients with Ulcerative Colitis
Source: PLoS One. 2015 Aug 19;10(8):e0133925. doi: 10.1371/journal.pone.0133925 (PMC4544847; doi:10.1371/journal.pone.0133925)
Supplement: S1 Questionnaire — (PDF) [file pone.0133925.s003.pdf]

FULL-LENGTH DONOR HISTORY QUESTIONNAIRE

Prescreen #:
Subject #:

RECEIVED
Human Subjects Division
DEC 05 2012
UW

DATE:		STAFF:	
AGE:	SEX:	HEIGHT:	WEIGHT:

		Yes	No
1. Do you live with the transplant recipient?		☐	☐
2. What is your relationship to transplant recipient?			
Are you:			
3. Feeling healthy and well today?		☐	☐
4. Currently taking an antibiotic or have you taken antibiotics in the past 12 weeks?		☐	☐
5. Currently taking any other medication for an infection?		☐	☐
In the past ***48 hours****:*			
6. Have you taken Aspirin or anything that has Aspirin in it? *- not an exclusion factor*		☐	☐
In the past ***two weeks:***			
7. Have you taken Steroids (including exogenous glucocorticoids)?		☐	☐
8. Have you taken Probiotics?		☐	☐
9. Have you taken Immunomodulators (including calcineurin inhibitors)?		☐	☐
10. Have you taken Biologics?		☐	☐
11. Taken any systemic antineoplastic agents (chemotherapy)?		☐	☐
12. *If subject has known food allergies:* Have you ingested (subject food allergen(s)) ____________ in the past two weeks?		☐	☐
Note washout period(s), if necessary:			
In the past ***6 weeks***:			
13. Female donors: Have you been pregnant or are you pregnant now? *- not an exclusion factor*	☐ I am male	☐	☐
In the past ***8 weeks****:*			
14. Have you had any vaccinations or other shots?		☐	☐
Have you had contact with someone who had a smallpox vaccination?		☐	☐

FULL-LENGTH DONOR HISTORY QUESTIONNAIRE

Prescreen #:
Subject #:

	Yes	No
*In the past **12 months** have you:*		
15. Had a transplant such as organ, tissue, or bone marrow?	☐	☐
16. Had a graft such as bone or skin?	☐	☐
17. Come into contact with someone else's blood?	☐	☐
18. Had an accidental needle-stick?	☐	☐
19. Had sexual contact with anyone who has HIV/AIDS or has had a positive test for the HIV/AIDS virus?	☐	☐
20. Had sexual contact with a prostitute or anyone else who takes money or drugs or other payment for sex?	☐	☐
21. Had sexual contact with anyone who has ever used needles to take drugs or steroids, or anything <u>not</u> prescribed by their doctor?	☐	☐
22. Had sexual contact with anyone who has hemophilia or has used clotting factor concentrates?	☐	☐
23. Female donors: Had sexual contact with a male who has ever had sexual contact with another male? ☐ I am male	☐	☐
24. Had sexual contact with a person who has hepatitis?	☐	☐
25. Lived with a person who had hepatitis?	☐	☐
26. ***Non-intimate donors:*** Had a tattoo?	☐	☐
27. ***Non-intimate donors:*** Had ear or body piercing?	☐	☐
28. Had or been treated for syphilis or gonorrhea?	☐	☐
29. ***Non-intimate donors:*** Been in juvenile detention, lockup, jail, or prison for more than 72 hours?	☐	☐

*In the past **three years** have you:*		
30. ***Non-intimate donors:*** Been outside the United States or Canada to countries where diarrheal illnesses are endemic or risk of traveler's diarrhea is high	☐	☐
*Location/Date of travel(s):*____________________________		

*From **1980 through 1996**:*		
31. Did you spend time that adds up to 3 months or more in the United Kingdom?	☐	☐
32. Were you a member of the U.S. military, a civilian military employee, or a dependent of a member of the U.S. military?	☐	☐

*From **1980 to the present**, did you:*		
33. Spend time that adds up to 5 years or more in Europe?	☐	☐

FULL-LENGTH DONOR HISTORY QUESTIONNAIRE

Prescreen #:
Subject #:

		Yes	No
*From **1977 to the present**, have you:*			
34. Received money, drugs, or other payment for sex?		☐	☐
35. Male donors: Had sexual contact with another male, even once?	☐ I am female	☐	☐

	Yes	No
*Have you **EVER**:*		
36. Had a positive test for the HIV/AIDS virus?	☐	☐
37. Used needles to take drugs, steroids, or anything not prescribed by your doctor?	☐	☐
38. Used clotting factor concentrates?	☐	☐
39. Had hepatitis?	☐	☐
40. Had malaria?	☐	☐
41. Had Chagas' disease?	☐	☐
42. Had babesiosis?	☐	☐
43. Received a dura mater (or brain covering) graft?	☐	☐
44. Had sexual contact with anyone who was born in or lived in Africa?	☐	☐
45. Had a relative with Creutzfeldt-Jakob disease?	☐	☐
46. Had inflammatory bowel disease?	☐	☐
47. Had irritable bowel syndrome, idiopathic chronic constipation, or chronic diarrhea? *Specify*________________	☐	☐
48. Had a gastrointestinal malignancy or colon polyps?	☐	☐

Relative Exclusion Criteria at researchers' discretion		
49. Have you ever had the infection *Clostridium difficile* ("C. Diff")?	☐	☐
50. Do you have a history of major gastrointestinal surgery? (e.g. gastric by-pass) *Specify*________________	☐	☐
51. Have you been diagnosed with an autoimmune disease? (e.g. multiple sclerosis, connective tissue disease) *Specify*________________	☐	☐
52. Have you been diagnosed with atopic diseases? (e.g. asthma, eczema, eosino-hilic disorder of the gastrointestinal tract) *Specify*________________	☐	☐
53. Have you been diagnosed with a chronic pain syndrome? (e.g chronic fatigue syndrome, fibromyalgia) *Specify*________________	☐	☐
54. Do you have diabetes and/or hypertension? *Specify*________________	☐	☐
55. Had any type of cancer, including leukemia? *Specify*________________	☐	☐
56. Had any problems with your heart or lungs? *Specify*________________	☐	☐
57. Had a bleeding condition or a blood disease? *Specify*________________	☐	☐
