# Supplementary material for: Low Level Engraftment and Improvement following a Single Colonoscopic Administration of Fecal Microbiota to Patients with Ulcerative Colitis
Source: PLoS One. 2015 Aug 19;10(8):e0133925. doi: 10.1371/journal.pone.0133925 (PMC4544847; doi:10.1371/journal.pone.0133925)
Supplement: S1 Table — (PDF) [file pone.0133925.s004.pdf]

S1I Table. Heatmap of Relative Species Abundance Across all Samples

classification	phylum	family	species
k__Archaea	Euryarchaeota	Methanobacteriaceae	Methanobrevibacter smithii
k__Archaea	Euryarchaeota	Methanobacteriaceae	Methanobrevibacter unclassified
k__Archaea	Euryarchaeota	Methanococcaceae	Methanococcus maripaludis
k__Archaea	Euryarchaeota	Methanococcaceae	Methanococcus unclassified
k__Bacteria	Acidobacteria	Acidobacteriaceae	Granulicella unclassified
k__Bacteria	Actinobacteria	Actinomycetaceae	***Actinomyces odontolyticus***
k__Bacteria	Actinobacteria	Actinomycetaceae	Actinomyces oris
k__Bacteria	Actinobacteria	Actinomycetaceae	Actinomyces viscosus
k__Bacteria	Actinobacteria	Micrococcaceae	Rothia mucilaginosa
k__Bacteria	Actinobacteria	Micrococcaceae	Rothia unclassified
k__Bacteria	Actinobacteria	Mycobacteriaceae	Mycobacterium unclassified
k__Bacteria	Actinobacteria	Propionibacteriaceae	Propionibacterium freudenreichii
k__Bacteria	Actinobacteria	Bifidobacteriaceae	**Bifidobacterium adolescentis**
k__Bacteria	Actinobacteria	Bifidobacteriaceae	Bifidobacterium animalis
k__Bacteria	Actinobacteria	Bifidobacteriaceae	Bifidobacterium bifidum
k__Bacteria	Actinobacteria	Bifidobacteriaceae	**Bifidobacterium breve**
k__Bacteria	Actinobacteria	Bifidobacteriaceae	**Bifidobacterium catenulatum**
k__Bacteria	Actinobacteria	Bifidobacteriaceae	**Bifidobacterium dentium**
k__Bacteria	Actinobacteria	Bifidobacteriaceae	Bifidobacterium longum
k__Bacteria	Actinobacteria	Bifidobacteriaceae	Bifidobacterium pseudocatenulatum
k__Bacteria	Actinobacteria	Bifidobacteriaceae	***Bifidobacterium unclassified***
k__Bacteria	Actinobacteria	Coriobacteriaceae	Atopobium parvulum
k__Bacteria	Actinobacteria	Coriobacteriaceae	Atopobium rimae
k__Bacteria	Actinobacteria	Coriobacteriaceae	Collinsella aerofaciens
k__Bacteria	Actinobacteria	Coriobacteriaceae	Collinsella intestinalis
k__Bacteria	Actinobacteria	Coriobacteriaceae	**Collinsella stercoris**
k__Bacteria	Actinobacteria	Coriobacteriaceae	**Eggerthella lenta**
k__Bacteria	Actinobacteria	Coriobacteriaceae	***Gordonibacter pamelaeae***
k__Bacteria	Actinobacteria	Coriobacteriaceae	**Olsenella uli**
k__Bacteria	Actinobacteria	Coriobacteriaceae	Slackia exigua
k__Bacteria	Actinobacteria	Coriobacteriaceae	Slackia unclassified
k__Bacteria	Bacteroidetes	Bacteroidaceae	Bacteroides caccae
k__Bacteria	Bacteroidetes	Bacteroidaceae	Bacteroides cellulosilyticus
k__Bacteria	Bacteroidetes	Bacteroidaceae	Bacteroides coprophilus
k__Bacteria	Bacteroidetes	Bacteroidaceae	Bacteroides dorei
k__Bacteria	Bacteroidetes	Bacteroidaceae	**Bacteroides eggerthii**
k__Bacteria	Bacteroidetes	Bacteroidaceae	Bacteroides finegoldii
k__Bacteria	Bacteroidetes	Bacteroidaceae	Bacteroides fragilis
k__Bacteria	Bacteroidetes	Bacteroidaceae	Bacteroides helcogenes
k__Bacteria	Bacteroidetes	Bacteroidaceae	Bacteroides intestinalis
k__Bacteria	Bacteroidetes	Bacteroidaceae	Bacteroides ovatus
k__Bacteria	Bacteroidetes	Bacteroidaceae	Bacteroides pectinophilus
k__Bacteria	Bacteroidetes	Bacteroidaceae	Bacteroides plebeius
k__Bacteria	Bacteroidetes	Bacteroidaceae	Bacteroides salanitronis
k__Bacteria	Bacteroidetes	Bacteroidaceae	Bacteroides stercoris
k__Bacteria	Bacteroidetes	Bacteroidaceae	***Bacteroides thetaiotaomicron***
k__Bacteria	Bacteroidetes	Bacteroidaceae	Bacteroides unclassified
k__Bacteria	Bacteroidetes	Bacteroidaceae	***Bacteroides uniformis***
k__Bacteria	Bacteroidetes	Bacteroidaceae	Bacteroides vulgatus
k__Bacteria	Bacteroidetes	Bacteroidaceae	Bacteroides xylanisolvens
k__Bacteria	Bacteroidetes	Porphyromonadaceae	Odoribacter splanchnicus
k__Bacteria	Bacteroidetes	Porphyromonadaceae	Parabacteroides distasonis
k__Bacteria	Bacteroidetes	Porphyromonadaceae	***Parabacteroides johnsonii***
k__Bacteria	Bacteroidetes	Porphyromonadaceae	***Parabacteroides merdae***
k__Bacteria	Bacteroidetes	Porphyromonadaceae	***Parabacteroides unclassified***
k__Bacteria	Bacteroidetes	Porphyromonadaceae	Porphyromonas asaccharolytica
k__Bacteria	Bacteroidetes	Porphyromonadaceae	**Porphyromonas uenonis**
k__Bacteria	Bacteroidetes	Prevotellaceae	Prevotella buccae
k__Bacteria	Bacteroidetes	Prevotellaceae	Prevotella buccalis
k__Bacteria	Bacteroidetes	Prevotellaceae	Prevotella copri
k__Bacteria	Bacteroidetes	Prevotellaceae	Prevotella disiens
k__Bacteria	Bacteroidetes	Prevotellaceae	Prevotella oris
k__Bacteria	Bacteroidetes	Rikenellaceae	Alistipes putredinis
k__Bacteria	Bacteroidetes	Rikenellaceae	***Alistipes shahii***
k__Bacteria	Bacteroidetes	Rikenellaceae	**Alistipes unclassified**
k__Bacteria	Bacteroidetes	Flavobacteriaceae	Cellulophaga unclassified
k__Bacteria	Bacteroidetes	Flavobacteriaceae	**Flavobacterium unclassified**
k__Bacteria	Firmicutes	Bacillales uncl	Gemella haemolysans
k__Bacteria	Firmicutes	Bacillales uncl	Gemella moribillum
k__Bacteria	Firmicutes	Bacillales uncl	***Gemella unclassified***
k__Bacteria	Firmicutes	Aerococcaceae	Abiotrophia defectiva
k__Bacteria	Firmicutes	Carnobacteriaceae	**Granulicatella adiacens**
k__Bacteria	Firmicutes	Carnobacteriaceae	***Granulicatella unclassified***
k__Bacteria	Firmicutes	Enterococcaceae	Enterococcus faecalis
k__Bacteria	Firmicutes	Enterococcaceae	Enterococcus faecium
k__Bacteria	Firmicutes	Enterococcaceae	Enterococcus unclassified
k__Bacteria	Firmicutes	Lactobacillaceae	Lactobacillus acidophilus
k__Bacteria	Firmicutes	Lactobacillaceae	Lactobacillus brevis
k__Bacteria	Firmicutes	Lactobacillaceae	Lactobacillus casei
k__Bacteria	Firmicutes	Lactobacillaceae	Lactobacillus delbrueckii
k__Bacteria	Firmicutes	Lactobacillaceae	Lactobacillus fermentum
k__Bacteria	Firmicutes	Lactobacillaceae	Lactobacillus gasseri
k__Bacteria	Firmicutes	Lactobacillaceae	Lactobacillus iners
k__Bacteria	Firmicutes	Lactobacillaceae	Lactobacillus paracasei
k__Bacteria	Firmicutes	Lactobacillaceae	Lactobacillus plantarum
k__Bacteria	Firmicutes	Lactobacillaceae	Lactobacillus rhamnosus
k__Bacteria	Firmicutes	Lactobacillaceae	Lactobacillus ruminis
k__Bacteria	Firmicutes	Lactobacillaceae	Lactobacillus sakei
k__Bacteria	Firmicutes	Lactobacillaceae	Pediococcus acidilactici
k__Bacteria	Firmicutes	Lactobacillaceae	Pediococcus pentosaceus
k__Bacteria	Firmicutes	Leuconostocaceae	Leuconostoc gasicomitatum
k__Bacteria	Firmicutes	Leuconostocaceae	Leuconostoc kimchii
k__Bacteria	Firmicutes	Leuconostocaceae	***Leuconostoc mesenteroides***
k__Bacteria	Firmicutes	Leuconostocaceae	Leuconostoc unclassified
k__Bacteria	Firmicutes	Streptococcaceae	Lactococcus lactis
k__Bacteria	Firmicutes	Streptococcaceae	Streptococcus agalactiae
k__Bacteria	Firmicutes	Streptococcaceae	Streptococcus anginosus
k__Bacteria	Firmicutes	Streptococcaceae	Streptococcus australis
k__Bacteria	Firmicutes	Streptococcaceae	Streptococcus cristatus
k__Bacteria	Firmicutes	Streptococcaceae	Streptococcus equinus
k__Bacteria	Firmicutes	Streptococcaceae	Streptococcus gordonii
k__Bacteria	Firmicutes	Streptococcaceae	Streptococcus infantarius
k__Bacteria	Firmicutes	Streptococcaceae	**Streptococcus infantis**
k__Bacteria	Firmicutes	Streptococcaceae	Streptococcus mitis
k__Bacteria	Firmicutes	Streptococcaceae	Streptococcus mutans
k__Bacteria	Firmicutes	Streptococcaceae	Streptococcus oralis
k__Bacteria	Firmicutes	Streptococcaceae	***Streptococcus parasanguinis***
k__Bacteria	Firmicutes	Streptococcaceae	Streptococcus pneumoniae
k__Bacteria	Firmicutes	Streptococcaceae	Streptococcus salivarius
k__Bacteria	Firmicutes	Streptococcaceae	**Streptococcus sanguinis**
k__Bacteria	Firmicutes	Streptococcaceae	Streptococcus thermophilus
k__Bacteria	Firmicutes	Streptococcaceae	**Streptococcus vestibularis**
k__Bacteria	Firmicutes	Clostridiaceae	***Alkaliphilus unclassified***
k__Bacteria	Firmicutes	Clostridiaceae	Clostridium asparagiforme
k__Bacteria	Firmicutes	Clostridiaceae	Clostridium bartlettii
k__Bacteria	Firmicutes	Clostridiaceae	Clostridium bolteae
k__Bacteria	Firmicutes	Clostridiaceae	**Clostridium botulinum**
k__Bacteria	Firmicutes	Clostridiaceae	**Clostridium cf**
k__Bacteria	Firmicutes	Clostridiaceae	Clostridium hathewayi
k__Bacteria	Firmicutes	Clostridiaceae	Clostridium hylemonae
k__Bacteria	Firmicutes	Clostridiaceae	**Clostridium leptum**
k__Bacteria	Firmicutes	Clostridiaceae	Clostridium methylpentosum
k__Bacteria	Firmicutes	Clostridiaceae	Clostridium nexile
k__Bacteria	Firmicutes	Clostridiaceae	Clostridium saccharolyticum
k__Bacteria	Firmicutes	Clostridiaceae	**Clostridium scindens**
k__Bacteria	Firmicutes	Clostridiaceae	Clostridium symbiosum
k__Bacteria	Firmicutes	Clostridiaceae	Pseudoflavonifractor capillosus
k__Bacteria	Firmicutes	Clostridiales Family XI Incertae Sedis	Anaerococcus prevotii
k__Bacteria	Firmicutes	Clostridiales Family XI Incertae Sedis	***Anaerococcus unclassified***
k__Bacteria	Firmicutes	Clostridiales Family XI Incertae Sedis	***Anaerococcus vaginalis***
k__Bacteria	Firmicutes	Clostridiales Family XI Incertae Sedis	Finegoldia magna
k__Bacteria	Firmicutes	Clostridiales Family XI Incertae Sedis	**Parvimonas micros**
k__Bacteria	Firmicutes	Clostridiales Family XI Incertae Sedis	Peptoniphilus duerdenii
k__Bacteria	Firmicutes	Clostridiales Family XI Incertae Sedis	Peptoniphilus harei
k__Bacteria	Firmicutes	Clostridiales Family XI Incertae Sedis	Peptoniphilus lacrimalis
k__Bacteria	Firmicutes	Clostridiales Family XI Incertae Sedis	Peptoniphilus unclassified
k__Bacteria	Firmicutes	Clostridiales uncl	**Blautia hansenii**
k__Bacteria	Firmicutes	Clostridiales uncl	***Blautia hydrogenotrophica***
k__Bacteria	Firmicutes	Clostridiales uncl	Blautia unclassified
k__Bacteria	Firmicutes	Eubacteriaceae	**Anaerofustis stercorihominis**
k__Bacteria	Firmicutes	Eubacteriaceae	**Eubacterium cellulosolvens**
k__Bacteria	Firmicutes	Eubacteriaceae	Eubacterium eligens
k__Bacteria	Firmicutes	Eubacteriaceae	Eubacterium hallii
k__Bacteria	Firmicutes	Eubacteriaceae	Eubacterium limosum
k__Bacteria	Firmicutes	Eubacteriaceae	**Eubacterium rectale**
k__Bacteria	Firmicutes	Eubacteriaceae	Eubacterium siraeum
k__Bacteria	Firmicutes	Eubacteriaceae	Eubacterium ventriosum
k__Bacteria	Firmicutes	Lachnospiraceae	Anaerostipes caccae
k__Bacteria	Firmicutes	Lachnospiraceae	Butyrivibrio crossotus
k__Bacteria	Firmicutes	Lachnospiraceae	Butyrivibrio unclassified
k__Bacteria	Firmicutes	Lachnospiraceae	Coprococcus catus
k__Bacteria	Firmicutes	Lachnospiraceae	Coprococcus comes
k__Bacteria	Firmicutes	Lachnospiraceae	**Coprococcus eutactus**
k__Bacteria	Firmicutes	Lachnospiraceae	***Dorea formicigenerans***
k__Bacteria	Firmicutes	Lachnospiraceae	Dorea longicatena
k__Bacteria	Firmicutes	Lachnospiraceae	Marvinbryantia formatexigens
k__Bacteria	Firmicutes	Lachnospiraceae	Oribacterium sinus
k__Bacteria	Firmicutes	Lachnospiraceae	***Roseburia intestinalis***
k__Bacteria	Firmicutes	Lachnospiraceae	Roseburia inulinivorans
k__Bacteria	Firmicutes	Lachnospiraceae	Roseburia unclassified
k__Bacteria	Firmicutes	Peptostreptococcaceae	Peptostreptococcus anaerobius
k__Bacteria	Firmicutes	Peptostreptococcaceae	***Peptostreptococcus stomatis***
k__Bacteria	Firmicutes	Peptostreptococcaceae	Peptostreptococcus unclassified
k__Bacteria	Firmicutes	Ruminococcaceae	***Anaerotruncus colihominis***
k__Bacteria	Firmicutes	Ruminococcaceae	***Faecalibacterium cf***
k__Bacteria	Firmicutes	Ruminococcaceae	***Faecalibacterium prausnitzii***
k__Bacteria	Firmicutes	Ruminococcaceae	***Faecalibacterium unclassified***
k__Bacteria	Firmicutes	Ruminococcaceae	Ruminococcus albus
k__Bacteria	Firmicutes	Ruminococcaceae	***Ruminococcus bromii***
k__Bacteria	Firmicutes	Ruminococcaceae	**Ruminococcus gnavus**
k__Bacteria	Firmicutes	Ruminococcaceae	***Ruminococcus lactaris***
k__Bacteria	Firmicutes	Ruminococcaceae	**Ruminococcus obeum**
k__Bacteria	Firmicutes	Ruminococcaceae	Ruminococcus torques
k__Bacteria	Firmicutes	Ruminococcaceae	Subdoligranulum variabile
k__Bacteria	Firmicutes	Erysipelotrichaceae	***Bulleidia extructa***
k__Bacteria	Firmicutes	Erysipelotrichaceae	Catenibacterium mitsuokai
k__Bacteria	Firmicutes	Erysipelotrichaceae	Coprobacillus bacterium
k__Bacteria	Firmicutes	Erysipelotrichaceae	Holdemania filiformis
k__Bacteria	Firmicutes	Erysipelotrichaceae	***Solobacterium moorei***
k__Bacteria	Firmicutes	Erysipelotrichaceae	Turicibacter unclassified
k__Bacteria	Firmicutes	Acidaminococcaceae	***Acidaminococcus fermentans***
k__Bacteria	Firmicutes	Veillonellaceae	Dialister invisus
k__Bacteria	Firmicutes	Veillonellaceae	Dialister microaerophilus
k__Bacteria	Firmicutes	Veillonellaceae	Megasphaera micronuciformis
k__Bacteria	Firmicutes	Veillonellaceae	Mitsuokella multacida
k__Bacteria	Firmicutes	Veillonellaceae	Selenomonas unclassified
k__Bacteria	Firmicutes	Veillonellaceae	Veillonella atypica
k__Bacteria	Firmicutes	Veillonellaceae	Veillonella dispar
k__Bacteria	Firmicutes	Veillonellaceae	***Veillonella parvula***
k__Bacteria	Firmicutes	Veillonellaceae	Veillonella unclassified
k__Bacteria	Fusobacteria	Fusobacteriaceae	**Fusobacterium nucleatum**
k__Bacteria	Lentisphaerae	Victivallaceae	Victivallis vadensis
k__Bacteria	Proteobacteria	Brucellaceae	Brucella suis
k__Bacteria	Proteobacteria	Burkholderiaceae	Burkholderia mallei
k__Bacteria	Proteobacteria	Burkholderiaceae	Burkholderia pseudomallei
k__Bacteria	Proteobacteria	Burkholderiaceae	Burkholderia unclassified
k__Bacteria	Proteobacteria	Oxalobacteraceae	Oxalobacter formigenes
k__Bacteria	Proteobacteria	Sutterellaceae	Sutterella wadsworthensis
k__Bacteria	Proteobacteria	Neisseriaceae	**Eikenella corrodens**
k__Bacteria	Proteobacteria	Neisseriaceae	Neisseria flavescens
k__Bacteria	Proteobacteria	Neisseriaceae	Neisseria unclassified
k__Bacteria	Proteobacteria	Desulfovibrionaceae	Bilophila wadsworthia
k__Bacteria	Proteobacteria	Desulfovibrionaceae	Desulfovibrio desulfuricans
k__Bacteria	Proteobacteria	Desulfovibrionaceae	Desulfovibrio piger
k__Bacteria	Proteobacteria	Enterobacteriaceae	Escherichia coli
k__Bacteria	Proteobacteria	Enterobacteriaceae	Escherichia unclassified
k__Bacteria	Proteobacteria	Enterobacteriaceae	Klebsiella pneumoniae
k__Bacteria	Proteobacteria	Enterobacteriaceae	Klebsiella unclassified
k__Bacteria	Proteobacteria	Enterobacteriaceae	Shigella boydii
k__Bacteria	Proteobacteria	Pasteurellaceae	**Aggregatibacter aphrophilus**
k__Bacteria	Proteobacteria	Pasteurellaceae	Aggregatibacter segnis
k__Bacteria	Proteobacteria	Pasteurellaceae	***Haemophilus parainfluenzae***
k__Bacteria	Proteobacteria	Pseudomonadaceae	Pseudomonas unclassified
k__Bacteria	Thermi	Deinococcaceae	***Deinococcus unclassified***
k__Bacteria	Verrucomicrobia	Verrucomicrobiaceae	Akkermansia muciniphila

Sample	Sample ID	6-Point UCDAI	Shannon Diveristy Inex
Donor 1 Sample 1	UCFMT1.D1.S1-2	0	2
Donor 1 Sample 2	UCFMT1.D1.S2-2	0	2
Recipient 1 Sample 1	UCFMT1.R1.S1-2	1	2
Recipient 1 Sample 2	UCFMT1.R1.S2-2	5	2
Recipient 1 Sample 3	UCFMT1.R1.S3-2	3	2
Recipient 1 Sample 4	UCFMT1.R1.S4-2	2	2
Recipient 1 Sample 5	UCFMT1.R1.S5	3	2
Donor 2 Sample 1	UCFMT1.D2.S1	0	2
Donor 2 Sample 2	UCFMT1.D2.S2	0	3
Recipient 2 Sample 1	UCFMT1.R2.S1-2	4	3
Recipient 2 Sample 2	UCFMT1.R2.S2	5	3
Recipient 2 Sample 3	UCFMT1.R2.S3-2	3	3
Recipient 2 Samle 4	UCFMT1.R2.S4	4	3
Recipient 2 Sample 5	UCFMT1.R2.S5	4	1
Donor 3 Sample 1	UCFMT1.D3.S1	0	3
Donor 3 Sample 2	UCFMT1.D3.S2	0	3
Recipient 3 Sample 1	UCFMT1.R3.S1	4	2
Recipient 3 Sample 2	UCFMT1.R3.S2	4	3
Recipient 3 Sample 3	UCFMT1.R3.S3	1	3
Recipient 3 Sample 4	UCFMT1.R3.S4	1	3
Recipient 3 Sample 5	UCFMT1.R3.S5	2	3
Donor 3 Sample 3	UCFMT1.D5.S3	0	3
Recipient 5 Sample 1	UCFMT1.R5.S1	2	3
Recipient 5 Sample 2	UCFMT1.R5.S2	3	3
Recipient 5 Sample 3	UCFMT1.R5.S3	2	3
Recipient 5 Sample 4	UCFMT1.R5.S4	2	3
Recipient 5 Sample 5	UCFMT1.R5.S5	2	3
Donor 6 Sample 1	UCFMT1.D6.S1	0	3
Donor 6 Sample 2	UCFMT1.D6.S2	0	2
Recipient 6 Sample 1	UCFMT1.R6.S1	3	1
Recipient 6 Sample 2	UCFMT1.R6.S2-2	4	3
Recipient 6 Sample 3	UCFMT1.R6.S3-2	1	3
Recipient 6 Sample 4	UCFMT1.R6.S4	5	2
Recipient 6 Sample 5	UCFMT1.R6.S5	4	3
Donor 7 Sample 1	UCFMT1.D7.S1-r	0	3
Donor 1 Sample 2	UCFMT1.D7.S2-r	0	2
Recipient 7 Sample 1	UCFMT1.R7.S1-r	3	3
Recipient 7 Sample 2	UCFMT1.R7.S2-r	5	3
Recipient 7 Sample 3	UCFMT1.R7.S3-r	4	3
Recipient 7 Sample 4	UCFMT1.R7.S4-r	4	3
Recipient 7 Sample 5	UCFMT1.R7.S5-r	5	3
Donor 8 Sample 1	UCFMT1.D8.S1	0	2
Donor 8 Sample 2	UCFMT1.D8.S2	0	3
Recipient 8 Sample 1	UCFMT1.R8.S1	4	3
Recipient 8 Sample 2	UCFMT1.R8.S2	5	2

Percent
100
10
1
0.1
0.01
0.001

Red font: all species that are higher in ulceraticve colitis at baseline.
Blue font: all species that are higher in health at baseline.
Green font: all species that are not detectable at baseline in either UC or healthy samples but that are detected at later timepoints.
