# Supplementary material for: Low Level Engraftment and Improvement following a Single Colonoscopic Administration of Fecal Microbiota to Patients with Ulcerative Colitis
Source: PLoS One. 2015 Aug 19;10(8):e0133925. doi: 10.1371/journal.pone.0133925 (PMC4544847; doi:10.1371/journal.pone.0133925)
Supplement: S2 Table — (PDF) [file pone.0133925.s005.pdf]

S2 Table. Differences in Percent Abundance of Species at Baseline Between Donor and Recipient

classification	phylum	family	species	Donor Average (D)	Recipient Average (R)	D-R	Log (D/R)	P Value*
k__Bacteria	Firmicutes	Ruminococcaceae	*Faecalibacterium unclassified*	0.554	2.217	-1.662	-0.602	0.04
k__Bacteria	Firmicutes	Ruminococcaceae	*Faecalibacterium cf*	1.326	2.739	-1.413	-0.315	0.02
k__Bacteria	Firmicutes	Lachnospiraceae	*Dorea formicigenerans*	0.948	1.660	-0.712	-0.243	0.02
k__Bacteria	Firmicutes	Clostridiales uncl	*Blautia hydrogenotrophica*	0.045	0.392	-0.348	-0.943	0.03
k__Bacteria	Bacteroidetes	Bacteroidaceae	*Bacteroides thetaiotaomicron*	0.011	0.147	-0.136	-1.143	0.04
k__Bacteria	Firmicutes	Erysipelotrichaceae	*Solobacterium moorei*	0.001	0.010	-0.009	-0.981	0.04
k__Bacteria	Actinobacteria	Coriobacteriaceae	*Gordonibacter pamelaeae*	0.151	0.042	0.108	0.553	0.04
k__Bacteria	Bacteroidetes	Porphyromonadaceae	*Parabacteroides johnsonii*	0.003	0.000	0.003	NA	0.04
k__Bacteria	Bacteroidetes	Porphyromonadaceae	*Parabacteroides merdae*	1.237	0.254	0.982	0.687	0.00
k__Bacteria	Bacteroidetes	Rikenellaceae	*Alistipes shahii*	0.497	0.190	0.307	0.417	0.04
k__Bacteria	Firmicutes	Clostridiaceae	*Alkaliphilus unclassified*	0.004	0.001	0.003	0.580	0.05
k__Bacteria	Firmicutes	Ruminococcaceae	*Anaerotruncus colihominis*	0.005	0.001	0.004	0.785	0.01
k__Bacteria	Firmicutes	Ruminococcaceae	*Ruminococcus bromii*	9.613	0.338	9.275	1.454	0.05
k__Bacteria	Firmicutes	Veillonellaceae	*Veillonella parvula*	0.009	0.001	0.009	1.132	0.05
k__Archaea	Euryarchaeota	Methanobacteriaceae	*Methanobrevibacter smithii*	0.002	0.467	-0.465	-2.345	0.35
k__Archaea	Euryarchaeota	Methanobacteriaceae	*Methanobrevibacter unclassified*	0.000	0.023	-0.023	-2.157	0.35
k__Archaea	Euryarchaeota	Methanococcaceae	*Methanococcus maripaludis*	0.000	0.000	0.000	NA	NA
k__Archaea	Euryarchaeota	Methanococcaceae	*Methanococcus unclassified*	0.000	0.000	0.000	NA	NA
k__Bacteria	Acidobacteria	Acidobacteriaceae	*Granulicella unclassified*	0.001	0.001	0.000	-0.079	0.87
k__Bacteria	Actinobacteria	Actinomycetaceae	*Actinomyces odontolyticus*	0.032	0.011	0.020	0.447	0.06
k__Bacteria	Actinobacteria	Actinomycetaceae	*Actinomyces oris*	0.001	0.000	0.001	0.539	0.39
k__Bacteria	Actinobacteria	Actinomycetaceae	*Actinomyces viscosus*	0.001	0.000	0.000	0.264	0.53
k__Bacteria	Actinobacteria	Micrococcaceae	*Rothia mucilaginosa*	0.001	0.007	-0.006	-0.741	0.28
k__Bacteria	Actinobacteria	Micrococcaceae	*Rothia unclassified*	0.000	0.001	-0.001	-0.711	0.34
k__Bacteria	Actinobacteria	Mycobacteriaceae	*Mycobacterium unclassified*	0.061	0.035	0.026	0.237	0.27
k__Bacteria	Actinobacteria	Propionibacteriaceae	*Propionibacterium freudenreichii*	0.006	0.005	0.002	0.128	0.83
k__Bacteria	Actinobacteria	Bifidobacteriaceae	*Bifidobacterium adolescentis*	8.914	3.909	5.005	0.358	0.15
k__Bacteria	Actinobacteria	Bifidobacteriaceae	*Bifidobacterium animalis*	0.003	0.998	-0.995	-2.495	0.33
k__Bacteria	Actinobacteria	Bifidobacteriaceae	*Bifidobacterium bifidum*	0.952	2.115	-1.163	-0.347	0.35
k__Bacteria	Actinobacteria	Bifidobacteriaceae	*Bifidobacterium breve*	0.000	0.013	-0.013	NA	0.16
k__Bacteria	Actinobacteria	Bifidobacteriaceae	*Bifidobacterium catenulatum*	0.015	0.003	0.012	0.685	0.15
k__Bacteria	Actinobacteria	Bifidobacteriaceae	*Bifidobacterium dentium*	0.000	0.027	-0.027	NA	0.15
k__Bacteria	Actinobacteria	Bifidobacteriaceae	*Bifidobacterium longum*	2.942	2.616	0.326	0.051	0.86
k__Bacteria	Actinobacteria	Bifidobacteriaceae	*Bifidobacterium pseudocatenulatum*	0.195	0.522	-0.326	-0.427	0.43
k__Bacteria	Actinobacteria	Bifidobacteriaceae	*Bifidobacterium unclassified*	0.029	0.004	0.026	0.889	0.09
k__Bacteria	Actinobacteria	Coriobacteriaceae	*Atopobium parvulum*	0.000	0.000	0.000	-0.051	0.94
k__Bacteria	Actinobacteria	Coriobacteriaceae	*Atopobium rimae*	0.000	0.000	0.000	NA	NA
k__Bacteria	Actinobacteria	Coriobacteriaceae	*Collinsella aerofaciens*	4.144	4.411	-0.267	-0.027	0.84
k__Bacteria	Actinobacteria	Coriobacteriaceae	*Collinsella intestinalis*	0.035	0.032	0.003	0.039	0.93
k__Bacteria	Actinobacteria	Coriobacteriaceae	*Collinsella stercoris*	0.078	0.000	0.078	2.721	0.14
k__Bacteria	Actinobacteria	Coriobacteriaceae	*Eggerthella lenta*	0.143	0.344	-0.201	-0.381	0.11
k__Bacteria	Actinobacteria	Coriobacteriaceae	*Olsenella uli*	0.000	0.000	0.000	NA	0.14
k__Bacteria	Actinobacteria	Coriobacteriaceae	*Slackia exigua*	0.000	0.000	0.000	NA	0.35
k__Bacteria	Actinobacteria	Coriobacteriaceae	*Slackia unclassified*	0.038	0.038	0.000	0.003	0.99
k__Bacteria	Bacteroidetes	Bacteroidaceae	*Bacteroides caccae*	0.182	0.102	0.080	0.253	0.37
k__Bacteria	Bacteroidetes	Bacteroidaceae	*Bacteroides cellulosilyticus*	0.038	0.067	-0.029	-0.246	0.57
k__Bacteria	Bacteroidetes	Bacteroidaceae	*Bacteroides coprophilus*	0.000	0.000	0.000	NA	NA
k__Bacteria	Bacteroidetes	Bacteroidaceae	*Bacteroides dorei*	0.086	0.036	0.051	0.383	0.25
k__Bacteria	Bacteroidetes	Bacteroidaceae	*Bacteroides eggerthii*	0.000	0.003	-0.003	-1.375	0.17
k__Bacteria	Bacteroidetes	Bacteroidaceae	*Bacteroides finegoldii*	0.150	0.028	0.121	0.721	0.26
k__Bacteria	Bacteroidetes	Bacteroidaceae	*Bacteroides fragilis*	0.748	0.315	0.434	0.376	0.42
k__Bacteria	Bacteroidetes	Bacteroidaceae	*Bacteroides helcogenes*	0.000	0.000	0.000	-0.160	0.81
k__Bacteria	Bacteroidetes	Bacteroidaceae	*Bacteroides intestinalis*	0.228	0.090	0.138	0.403	0.44
k__Bacteria	Bacteroidetes	Bacteroidaceae	*Bacteroides ovatus*	0.521	0.669	-0.149	-0.109	0.69
k__Bacteria	Bacteroidetes	Bacteroidaceae	*Bacteroides pectinophilus*	0.000	0.000	0.000	NA	0.31
k__Bacteria	Bacteroidetes	Bacteroidaceae	*Bacteroides plebeius*	0.022	0.133	-0.111	-0.780	0.27
k__Bacteria	Bacteroidetes	Bacteroidaceae	*Bacteroides salanitronis*	0.000	0.000	0.000	-0.665	0.29
k__Bacteria	Bacteroidetes	Bacteroidaceae	*Bacteroides stercoris*	2.243	2.663	-0.421	-0.075	0.80
k__Bacteria	Bacteroidetes	Bacteroidaceae	*Bacteroides unclassified*	5.181	8.218	-3.037	-0.200	0.41
k__Bacteria	Bacteroidetes	Bacteroidaceae	*Bacteroides uniformis*	0.346	0.127	0.220	0.437	0.08
k__Bacteria	Bacteroidetes	Bacteroidaceae	*Bacteroides vulgatus*	7.520	9.328	-1.807	-0.094	0.71
k__Bacteria	Bacteroidetes	Bacteroidaceae	*Bacteroides xylanisolvens*	0.495	0.548	-0.053	-0.044	0.83
k__Bacteria	Bacteroidetes	Porphyromonadaceae	*Odoribacter splanchnicus*	0.431	0.342	0.088	0.099	0.54
k__Bacteria	Bacteroidetes	Porphyromonadaceae	*Parabacteroides distasonis*	0.021	0.012	0.009	0.230	0.60
k__Bacteria	Bacteroidetes	Porphyromonadaceae	*Parabacteroides unclassified*	0.150	0.039	0.111	0.584	0.06
k__Bacteria	Bacteroidetes	Porphyromonadaceae	*Porphyromonas asaccharolytica*	0.000	0.003	-0.003	NA	0.21
k__Bacteria	Bacteroidetes	Porphyromonadaceae	*Porphyromonas uenonis*	0.000	0.004	-0.004	NA	0.18
k__Bacteria	Bacteroidetes	Prevotellaceae	*Prevotella buccae*	0.000	0.034	-0.034	NA	0.35
k__Bacteria	Bacteroidetes	Prevotellaceae	*Prevotella buccalis*	0.000	0.001	-0.001	NA	0.29
k__Bacteria	Bacteroidetes	Prevotellaceae	*Prevotella copri*	0.264	1.126	-0.862	-0.629	0.45
k__Bacteria	Bacteroidetes	Prevotellaceae	*Prevotella disiens*	0.000	0.000	0.000	NA	0.35
k__Bacteria	Bacteroidetes	Prevotellaceae	*Prevotella oris*	0.000	0.000	0.000	NA	NA
k__Bacteria	Bacteroidetes	Rikenellaceae	*Alistipes putredinis*	1.976	1.916	0.060	0.013	0.96
k__Bacteria	Bacteroidetes	Rikenellaceae	*Alistipes unclassified*	0.000	0.036	-0.036	NA	0.18
k__Bacteria	Bacteroidetes	Flavobacteriaceae	*Cellulophaga unclassified*	0.000	0.000	0.000	NA	0.31
k__Bacteria	Bacteroidetes	Flavobacteriaceae	*Flavobacterium unclassified*	0.002	0.000	0.002	0.775	0.11
k__Bacteria	Firmicutes	Bacillales uncl	*Gemella haemolysans*	0.000	0.000	0.000	NA	NA
k__Bacteria	Firmicutes	Bacillales uncl	*Gemella moribillum*	0.000	3.241	-3.241	NA	0.32
k__Bacteria	Firmicutes	Bacillales uncl	*Gemella unclassified*	0.000	0.008	-0.008	-2.130	0.06
k__Bacteria	Firmicutes	Aerococcaceae	*Abiotrophia defectiva*	0.000	0.000	0.000	NA	0.35
k__Bacteria	Firmicutes	Carnobacteriaceae	*Granulicatella adiacens*	0.001	0.014	-0.014	-1.451	0.18
k__Bacteria	Firmicutes	Carnobacteriaceae	*Granulicatella unclassified*	0.001	0.005	-0.004	-0.552	0.10
k__Bacteria	Firmicutes	Enterococcaceae	*Enterococcus faecalis*	0.000	0.002	-0.002	NA	0.35
k__Bacteria	Firmicutes	Enterococcaceae	*Enterococcus faecium*	0.000	0.255	-0.255	NA	0.35
k__Bacteria	Firmicutes	Enterococcaceae	*Enterococcus unclassified*	0.006	0.276	-0.270	-1.672	0.34
k__Bacteria	Firmicutes	Lactobacillaceae	*Lactobacillus acidophilus*	0.000	0.003	-0.003	-1.729	0.24
k__Bacteria	Firmicutes	Lactobacillaceae	*Lactobacillus brevis*	0.001	0.000	0.001	NA	0.31
k__Bacteria	Firmicutes	Lactobacillaceae	*Lactobacillus casei*	0.000	0.000	0.000	0.798	0.40
k__Bacteria	Firmicutes	Lactobacillaceae	*Lactobacillus delbrueckii*	0.000	0.001	0.000	-0.227	0.72
k__Bacteria	Firmicutes	Lactobacillaceae	*Lactobacillus fermentum*	0.000	0.000	0.000	NA	NA
k__Bacteria	Firmicutes	Lactobacillaceae	*Lactobacillus gasseri*	0.000	0.001	-0.001	NA	0.35
k__Bacteria	Firmicutes	Lactobacillaceae	*Lactobacillus iners*	0.000	0.000	0.000	NA	NA
k__Bacteria	Firmicutes	Lactobacillaceae	*Lactobacillus paracasei*	0.000	0.000	0.000	NA	0.31
k__Bacteria	Firmicutes	Lactobacillaceae	*Lactobacillus plantarum*	0.000	0.002	-0.002	NA	0.35
k__Bacteria	Firmicutes	Lactobacillaceae	*Lactobacillus rhamnosus*	0.003	0.001	0.002	0.562	0.47
k__Bacteria	Firmicutes	Lactobacillaceae	*Lactobacillus ruminis*	0.238	0.000	0.238	NA	0.27
k__Bacteria	Firmicutes	Lactobacillaceae	*Lactobacillus sakei*	0.045	0.007	0.037	0.791	0.40
k__Bacteria	Firmicutes	Lactobacillaceae	*Pediococcus acidilactici*	0.001	0.000	0.001	NA	0.22
k__Bacteria	Firmicutes	Lactobacillaceae	*Pediococcus pentosaceus*	0.011	0.000	0.011	NA	0.31
k__Bacteria	Firmicutes	Leuconostocaceae	*Leuconostoc gasicomitatum*	0.001	0.005	-0.003	-0.628	0.34
k__Bacteria	Firmicutes	Leuconostocaceae	*Leuconostoc kimchii*	0.000	0.006	-0.006	NA	0.35
k__Bacteria	Firmicutes	Leuconostocaceae	*Leuconostoc mesenteroides*	0.000	0.002	-0.002	NA	0.09
k__Bacteria	Firmicutes	Leuconostocaceae	*Leuconostoc unclassified*	0.000	0.003	-0.003	NA	0.35
k__Bacteria	Firmicutes	Streptococcaceae	*Lactococcus lactis*	0.023	0.109	-0.086	-0.679	0.30
k__Bacteria	Firmicutes	Streptococcaceae	*Streptococcus agalactiae*	0.000	0.000	0.000	NA	0.31
k__Bacteria	Firmicutes	Streptococcaceae	*Streptococcus anginosus*	0.001	0.745	-0.744	-3.093	0.33
k__Bacteria	Firmicutes	Streptococcaceae	*Streptococcus australis*	0.047	0.080	-0.033	-0.231	0.47
k__Bacteria	Firmicutes	Streptococcaceae	*Streptococcus cristatus*	0.000	0.000	0.000	0.017	0.98
k__Bacteria	Firmicutes	Streptococcaceae	*Streptococcus equinus*	0.000	0.000	0.000	NA	NA
k__Bacteria	Firmicutes	Streptococcaceae	*Streptococcus gordonii*	0.001	0.000	0.001	1.585	0.30
k__Bacteria	Firmicutes	Streptococcaceae	*Streptococcus infantarius*	0.000	0.000	0.000	NA	NA
k__Bacteria	Firmicutes	Streptococcaceae	*Streptococcus infantis*	0.002	0.009	-0.007	-0.698	0.13
k__Bacteria	Firmicutes	Streptococcaceae	*Streptococcus mitis*	0.012	0.017	-0.005	-0.157	0.53
k__Bacteria	Firmicutes	Streptococcaceae	*Streptococcus mutans*	0.000	0.000	0.000	-0.380	0.61
k__Bacteria	Firmicutes	Streptococcaceae	*Streptococcus oralis*	0.000	0.001	0.000	-0.425	0.50
k__Bacteria	Firmicutes	Streptococcaceae	*Streptococcus parasanguinis*	0.045	0.140	-0.095	-0.497	0.09
k__Bacteria	Firmicutes	Streptococcaceae	*Streptococcus pneumoniae*	0.000	0.000	0.000	NA	0.31
k__Bacteria	Firmicutes	Streptococcaceae	*Streptococcus salivarius*	0.137	0.281	-0.144	-0.311	0.29
k__Bacteria	Firmicutes	Streptococcaceae	*Streptococcus sanguinis*	0.001	0.008	-0.007	-0.915	0.17
k__Bacteria	Firmicutes	Streptococcaceae	*Streptococcus thermophilus*	0.177	0.171	0.006	0.015	0.95
k__Bacteria	Firmicutes	Streptococcaceae	*Streptococcus vestibularis*	0.002	0.011	-0.010	-0.844	0.12
k__Bacteria	Firmicutes	Clostridiaceae	*Clostridium asparagiforme*	0.014	0.014	-0.001	-0.023	0.95
k__Bacteria	Firmicutes	Clostridiaceae	*Clostridium bartlettii*	0.180	0.189	-0.009	-0.021	0.96
k__Bacteria	Firmicutes	Clostridiaceae	*Clostridium bolteae*	0.031	0.027	0.004	0.059	0.86
k__Bacteria	Firmicutes	Clostridiaceae	*Clostridium botulinum*	0.000	0.001	-0.001	-1.017	0.20
k__Bacteria	Firmicutes	Clostridiaceae	*Clostridium cf*	0.011	0.033	-0.023	-0.499	0.17
k__Bacteria	Firmicutes	Clostridiaceae	*Clostridium hathewayi*	0.012	0.011	0.001	0.033	0.93
k__Bacteria	Firmicutes	Clostridiaceae	*Clostridium hylemonae*	0.000	0.000	0.000	NA	0.31
k__Bacteria	Firmicutes	Clostridiaceae	*Clostridium leptum*	0.380	0.014	0.365	1.419	0.15
k__Bacteria	Firmicutes	Clostridiaceae	*Clostridium methylpentosum*	0.001	0.000	0.001	0.325	0.37
k__Bacteria	Firmicutes	Clostridiaceae	*Clostridium nexile*	0.219	0.059	0.160	0.571	0.30
k__Bacteria	Firmicutes	Clostridiaceae	*Clostridium saccharolyticum*	0.000	0.000	0.000	0.489	0.25
k__Bacteria	Firmicutes	Clostridiaceae	*Clostridium scindens*	0.028	0.007	0.021	0.619	0.15
k__Bacteria	Firmicutes	Clostridiaceae	*Clostridium symbiosum*	0.031	0.087	-0.056	-0.449	0.39
k__Bacteria	Firmicutes	Clostridiaceae	*Pseudoflavonifractor capillosus*	0.054	0.030	0.024	0.257	0.22
k__Bacteria	Firmicutes	Clostridiales Family XI Incertae Sedis	*Anaerococcus prevotii*	0.000	0.000	0.000	NA	0.35
k__Bacteria	Firmicutes	Clostridiales Family XI Incertae Sedis	*Anaerococcus unclassified*	0.002	0.095	-0.093	-1.673	0.08
k__Bacteria	Firmicutes	Clostridiales Family XI Incertae Sedis	*Anaerococcus vaginalis*	0.000	0.022	-0.022	NA	0.08
k__Bacteria	Firmicutes	Clostridiales Family XI Incertae Sedis	*Finegoldia magna*	0.000	0.002	-0.002	NA	0.30
k__Bacteria	Firmicutes	Clostridiales Family XI Incertae Sedis	*Parvimonas micros*	0.000	0.117	-0.117	-2.721	0.15
k__Bacteria	Firmicutes	Clostridiales Family XI Incertae Sedis	*Peptoniphilus duerdenii*	0.000	0.000	0.000	NA	NA
k__Bacteria	Firmicutes	Clostridiales Family XI Incertae Sedis	*Peptoniphilus harei*	0.000	0.182	-0.182	NA	0.26
k__Bacteria	Firmicutes	Clostridiales Family XI Incertae Sedis	*Peptoniphilus lacrimalis*	0.000	0.000	0.000	NA	0.35
k__Bacteria	Firmicutes	Clostridiales Family XI Incertae Sedis	*Peptoniphilus unclassified*	0.046	0.686	-0.640	-1.174	0.32
k__Bacteria	Firmicutes	Clostridiales uncl	*Blautia hansenii*	0.797	0.027	0.770	1.477	0.15
k__Bacteria	Firmicutes	Clostridiales uncl	*Blautia unclassified*	1.057	1.455	-0.398	-0.139	0.37
k__Bacteria	Firmicutes	Eubacteriaceae	*Anaerofustis stercorihominis*	0.000	0.000	0.000	NA	0.14
k__Bacteria	Firmicutes	Eubacteriaceae	*Eubacterium cellulosolvens*	0.000	0.000	0.000	NA	0.16
k__Bacteria	Firmicutes	Eubacteriaceae	*Eubacterium eligens*	1.717	3.114	-1.396	-0.258	0.27
k__Bacteria	Firmicutes	Eubacteriaceae	*Eubacterium hallii*	1.397	1.015	0.383	0.139	0.34
k__Bacteria	Firmicutes	Eubacteriaceae	*Eubacterium limosum*	0.000	0.003	-0.003	NA	0.21
k__Bacteria	Firmicutes	Eubacteriaceae	*Eubacterium rectale*	14.728	6.885	7.843	0.330	0.11
k__Bacteria	Firmicutes	Eubacteriaceae	*Eubacterium siraeum*	0.563	0.975	-0.412	-0.239	0.70
k__Bacteria	Firmicutes	Eubacteriaceae	*Eubacterium ventriosum*	0.281	0.275	0.006	0.010	0.97
k__Bacteria	Firmicutes	Lachnospiraceae	*Anaerostipes caccae*	0.001	0.001	0.000	-0.125	0.78
k__Bacteria	Firmicutes	Lachnospiraceae	*Butyrivibrio crossotus*	0.020	0.000	0.020	NA	0.31
k__Bacteria	Firmicutes	Lachnospiraceae	*Butyrivibrio unclassified*	0.077	0.081	-0.004	-0.022	0.86
k__Bacteria	Firmicutes	Lachnospiraceae	*Coprococcus catus*	0.607	0.615	-0.008	-0.006	0.97
k__Bacteria	Firmicutes	Lachnospiraceae	*Coprococcus comes*	2.131	3.104	-0.972	-0.163	0.35
k__Bacteria	Firmicutes	Lachnospiraceae	*Coprococcus eutactus*	0.000	0.000	0.000	NA	0.20
k__Bacteria	Firmicutes	Lachnospiraceae	*Dorea longicatena*	2.671	2.792	-0.121	-0.019	0.86
k__Bacteria	Firmicutes	Lachnospiraceae	*Marvinbryantia formatexigens*	0.002	0.028	-0.026	-1.223	0.37
k__Bacteria	Firmicutes	Lachnospiraceae	*Oribacterium sinus*	0.000	0.000	0.000	1.163	0.29
k__Bacteria	Firmicutes	Lachnospiraceae	*Roseburia intestinalis*	2.737	0.367	2.369	0.872	0.10
k__Bacteria	Firmicutes	Lachnospiraceae	*Roseburia inulinivorans*	0.987	1.035	-0.048	-0.021	0.91
k__Bacteria	Firmicutes	Lachnospiraceae	*Roseburia unclassified*	0.000	0.000	0.000	NA	NA
k__Bacteria	Firmicutes	Peptostreptococcaceae	*Peptostreptococcus anaerobius*	0.000	0.167	-0.167	NA	0.33
k__Bacteria	Firmicutes	Peptostreptococcaceae	*Peptostreptococcus stomatis*	0.001	0.066	-0.066	-1.930	0.10
k__Bacteria	Firmicutes	Peptostreptococcaceae	*Peptostreptococcus unclassified*	0.001	0.001	0.000	-0.132	0.82
k__Bacteria	Firmicutes	Ruminococcaceae	*Faecalibacterium prausnitzii*	5.678	10.075	-4.397	-0.249	0.11
k__Bacteria	Firmicutes	Ruminococcaceae	*Ruminococcus albus*	0.003	0.005	-0.002	-0.215	0.47
k__Bacteria	Firmicutes	Ruminococcaceae	*Ruminococcus gnavus*	1.186	0.268	0.918	0.646	0.18
k__Bacteria	Firmicutes	Ruminococcaceae	*Ruminococcus lactaris*	1.891	0.608	1.282	0.492	0.06
k__Bacteria	Firmicutes	Ruminococcaceae	*Ruminococcus obeum*	1.712	2.906	-1.194	-0.230	0.13
k__Bacteria	Firmicutes	Ruminococcaceae	*Ruminococcus torques*	1.403	1.880	-0.477	-0.127	0.49
k__Bacteria	Firmicutes	Ruminococcaceae	*Subdoligranulum variabile*	0.051	0.063	-0.012	-0.088	0.58
k__Bacteria	Firmicutes	Erysipelotrichaceae	*Bulleidia extructa*	0.001	0.000	0.001	NA	0.08
k__Bacteria	Firmicutes	Erysipelotrichaceae	*Catenibacterium mitsuokai*	0.130	0.106	0.024	0.088	0.87
k__Bacteria	Firmicutes	Erysipelotrichaceae	*Coprobacillus bacterium*	0.267	0.095	0.172	0.448	0.35
k__Bacteria	Firmicutes	Erysipelotrichaceae	*Holdemania filiformis*	0.063	0.072	-0.009	-0.058	0.78
k__Bacteria	Firmicutes	Erysipelotrichaceae	*Turicibacter unclassified*	0.012	0.050	-0.037	-0.610	0.35
k__Bacteria	Firmicutes	Acidaminococcaceae	*Acidaminococcus fermentans*	0.000	0.000	0.000	NA	0.10
k__Bacteria	Firmicutes	Veillonellaceae	*Dialister invisus*	1.709	0.630	1.079	0.434	0.23
k__Bacteria	Firmicutes	Veillonellaceae	*Dialister microaerophilus*	0.000	0.001	-0.001	NA	0.35
k__Bacteria	Firmicutes	Veillonellaceae	*Megasphaera micronuciformis*	0.000	0.000	0.000	NA	0.31
k__Bacteria	Firmicutes	Veillonellaceae	*Mitsuokella multacida*	0.044	0.000	0.044	NA	0.31
k__Bacteria	Firmicutes	Veillonellaceae	*Selenomonas unclassified*	0.001	0.000	0.000	0.191	0.62
k__Bacteria	Firmicutes	Veillonellaceae	*Veillonella atypica*	0.021	0.003	0.018	0.859	0.27
k__Bacteria	Firmicutes	Veillonellaceae	*Veillonella dispar*	0.000	0.000	0.000	-0.121	0.78
k__Bacteria	Firmicutes	Veillonellaceae	*Veillonella unclassified*	0.011	0.007	0.004	0.199	0.57
k__Bacteria	Fusobacteria	Fusobacteriaceae	*Fusobacterium nucleatum*	0.000	0.001	-0.001	NA	0.17
k__Bacteria	Lentisphaerae	Victivallaceae	*Victivallis vadensis*	0.001	0.000	0.001	NA	0.31
k__Bacteria	Proteobacteria	Brucellaceae	*Brucella suis*	0.000	0.000	0.000	NA	NA
k__Bacteria	Proteobacteria	Burkholderiaceae	*Burkholderia mallei*	0.000	0.000	0.000	NA	NA
k__Bacteria	Proteobacteria	Burkholderiaceae	*Burkholderia pseudomallei*	0.000	0.000	0.000	NA	NA
k__Bacteria	Proteobacteria	Burkholderiaceae	*Burkholderia unclassified*	0.001	0.001	0.000	-0.014	0.97
k__Bacteria	Proteobacteria	Oxalobacteraceae	*Oxalobacter formigenes*	0.004	0.000	0.004	NA	0.23
k__Bacteria	Proteobacteria	Sutterellaceae	*Sutterella wadsworthensis*	1.008	1.265	-0.256	-0.098	0.68
k__Bacteria	Proteobacteria	Neisseriaceae	*Eikenella corrodens*	0.000	0.006	-0.006	NA	0.19
k__Bacteria	Proteobacteria	Neisseriaceae	*Neisseria flavescens*	0.000	0.000	0.000	NA	0.35
k__Bacteria	Proteobacteria	Neisseriaceae	*Neisseria unclassified*	0.000	0.000	0.000	NA	NA
k__Bacteria	Proteobacteria	Desulfovibrionaceae	*Bilophila wadsworthia*	0.183	0.146	0.037	0.097	0.73
k__Bacteria	Proteobacteria	Desulfovibrionaceae	*Desulfovibrio desulfuricans*	0.077	0.045	0.032	0.235	0.24
k__Bacteria	Proteobacteria	Desulfovibrionaceae	*Desulfovibrio piger*	0.000	0.000	0.000	NA	0.31
k__Bacteria	Proteobacteria	Enterobacteriaceae	*Escherichia coli*	0.025	2.810	-2.785	-2.048	0.27
k__Bacteria	Proteobacteria	Enterobacteriaceae	*Escherichia unclassified*	0.001	0.000	0.001	NA	0.31
k__Bacteria	Proteobacteria	Enterobacteriaceae	*Klebsiella pneumoniae*	0.000	0.002	-0.002	NA	0.35
k__Bacteria	Proteobacteria	Enterobacteriaceae	*Klebsiella unclassified*	0.000	0.000	0.000	NA	0.35
k__Bacteria	Proteobacteria	Enterobacteriaceae	*Shigella boydii*	0.000	0.000	0.000	NA	NA
k__Bacteria	Proteobacteria	Pasteurellaceae	*Aggregatibacter aphrophilus*	0.000	0.003	-0.003	NA	0.18
k__Bacteria	Proteobacteria	Pasteurellaceae	*Aggregatibacter segnis*	0.000	0.120	-0.120	NA	0.35
k__Bacteria	Proteobacteria	Pasteurellaceae	*Haemophilus parainfluenzae*	0.005	0.208	-0.203	-1.612	0.09
k__Bacteria	Proteobacteria	Pseudomonadaceae	*Pseudomonas unclassified*	0.001	0.001	-0.001	-0.284	0.59
k__Bacteria	Thermi	Deinococcaceae	*Deinococcus unclassified*	0.005	0.001	0.004	0.688	0.09
k__Bacteria	Verrucomicrobia	Verrucomicrobiaceae	*Akkermansia muciniphila*	0.092	0.002	0.090	1.669	0.31

Sample	Sample ID	6-Point UCDAI	Shannon Diversity Index
Donor 1 Sample 1	UCFMT1.D1.S1-2	0	2
Donor 1 Sample 2	UCFMT1.D1.S2-2	0	2
Donor 2 Sample 1	UCFMT1.D2.S1	0	2
Donor 2 Sample 2	UCFMT1.D2.S2	0	3
Donor 3 Sample 1	UCFMT1.D3.S1	0	3
Donor 3 Sample 2	UCFMT1.D3.S2	0	3
Donor 3 Sample 3	UCFMT1.D5.S3	0	3
Donor 6 Sample 1	UCFMT1.D6.S1	0	3
Donor 6 Sample 2	UCFMT1.D6.S2	0	2
Donor 7 Sample 1	UCFMT1.D7.S1-r	0	3
Donor 7 Sample 2	UCFMT1.D7.S2-r	0	2
Donor 8 Sample 1	UCFMT1.D8.S1	0	2
Donor 8 Sample 2	UCFMT1.D8.S2	0	3
Recipient 1 Sample 1	UCFMT1.R1.S1-2	1	2
Recipient 1 Sample 2	UCFMT1.R1.S2-2	5	2
Recipient 2 Sample 1	UCFMT1.R2.S1-2	4	3
Recipient 2 Sample 2	UCFMT1.R2.S2	5	3
Recipient 3 Sample 1	UCFMT1.R3.S1	4	2
Recipient 3 Sample 2	UCFMT1.R3.S2	4	3
Recipient 5 Sample 1	UCFMT1.R5.S1	2	3
Recipient 5 Sample 2	UCFMT1.R5.S2	3	3
Recipient 6 Sample 1	UCFMT1.R6.S1	3	1
Recipient 6 Sample 2	UCFMT1.R6.S2-2	4	3
Recipient 7 Sample 1	UCFMT1.R7.S1-r	3	3
Recipient 7 Sample 2	UCFMT1.R7.S2-r	5	3
Recipient 8 Sample 1	UCFMT1.R8.S1	4	3
Recipient 8 Sample 2	UCFMT1.R8.S2	5	2

Percent
100
10
1
0.1
0.01
0.001

*Please note these p values do not inclue statistical correction for multiple testing and are considered exploratory. When correction for multiple testing is performed the differences are not statistically signficant.

Species defined as "enriched" are present with uncorrected p values of less than 0.05 and are located above the black line.

Red font: all species that are higher in ulceraticve colitis at baseline.

Blue font: all species that are higher in health at baseline.

Green font: all species that are not detectable at baseline in either UC or healthy samples but that are detected at later timepoints.

NA: not applicable
