# Supplementary material for: Low Level Engraftment and Improvement following a Single Colonoscopic Administration of Fecal Microbiota to Patients with Ulcerative Colitis
Source: PLoS One. 2015 Aug 19;10(8):e0133925. doi: 10.1371/journal.pone.0133925 (PMC4544847; doi:10.1371/journal.pone.0133925)
Supplement: S3 Table — (PDF) [file pone.0133925.s006.pdf]

S3 Table. Relative and Absolute Changes in Percent Abundance of Species

				Absolute Change (Percent Abundance$_x$-Percent Abundance$_{BL2}$) (%)																		Relative Change (log_{10}(Percent Abundance$_x$/Percent Abundance$_{BL2}$))																	
classification	phylum	family	species	Recipient 1 Sample 3	Recipient 1 Sample 4	Recipient 1 Sample 5	Recipient 2 Sample 3	Recipient 2 Sample 4	Recipient 2 Sample 5	Recipient 3 Sample 3	Recipient 3 Sample 4	Recipient 3 Sample 5	Recipient 5 Sample 3	Recipient 5 Sample 4	Recipient 5 Sample 5	Recipient 6 sAmple 3	Recipient 6 Sample 4	Recipient 6 Sample 5	Recipient 7 Sample 3	Recipient 7 Sample 4	Recipient 7 Sample 5	Recipient 1 Sample 3	Recipient 1 Sample 4	Recipient 1 Sample 5	Recipient 2 Sample 3	Recipient 2 Sample 4	Recipient 2 Sample 5	Recipient 3 Sample 3	Recipient 3 Sample 4	Recipient 3 Sample 5	Recipient 5 Sample 3	Recipient 5 Sample 4	Recipient 5 Sample 5	Recipient 6 sAmple 3	Recipient 6 Sample 4	Recipient 6 Sample 5	Recipient 7 Sample 3	Recipient 7 Sample 4	Recipient 7 Sample 5
k__Archaea	Euryarchaeota	Methanobacteriaceae	Methanobrevibacter smithii				0						0			0.83	-5.2	-2.6							∞						∞			0.05	-0.7	-0.2			
k__Archaea	Euryarchaeota	Methanobacteriaceae	Methanobrevibacter unclassified													-0.1	-0.3	-0.1																-0.1	-0.7	-0.2			
k__Archaea	Euryarchaeota	Methanococcaceae	Methanococcus maripaludis																																				
k__Archaea	Euryarchaeota	Methanococcaceae	Methanococcus unclassified																																				
k__Bacteria	Acidobacteria	Acidobacteriaceae	Granulicella unclassified													-0	-0	0	-0	-0	-0													-∞	-0.2	0.26	-∞	-∞	-∞
k__Bacteria	Actinobacteria	Actinomycetaceae	Actinomyces odontolyticus	-0	0	0	0	-0	-0	0.02	-0	-0	0.01	0	0.01	-0	-0	-0	-0	-0	-0	-0.4	0.34	0.31	0.1	-0.9	-∞	0.4	-0.2	-∞	1.15	0.49	0.87	-0.2	-0.3	-0.5	-0.5	-0.4	-0.3
k__Bacteria	Actinobacteria	Actinomycetaceae	Actinomyces oris																-0	-0	-0																-∞	-∞	-0.2
k__Bacteria	Actinobacteria	Actinomycetaceae	Actinomyces viscosus			0	-0	-0	-0							-0	-0	-0	-0	-0	0			∞	-∞	-∞	-∞							-∞	-∞	-∞	-∞	-∞	0.06
k__Bacteria	Actinobacteria	Micrococcaceae	Rothia mucilaginosa				-0	-0.1	-0.1	-0	-0	-0					0	0							-0.5	-∞	-∞	-∞	-1	-∞					∞	∞			
k__Bacteria	Actinobacteria	Micrococcaceae	Rothia unclassified				-0	-0	-0	0							0	0							-∞	-∞	-∞	∞							-∞	∞			
k__Bacteria	Actinobacteria	Mycobacteriaceae	Mycobacterium unclassified	-0	0.08	0.1	0.01	0	-0	-0	-0	0.04	0.01	0	0.01	0.09	0.02	0.02	-0	0.01	0.01	-∞	0.66	0.73	0.23	0.11	-∞	-0.4	-0.8	0.42	∞	∞	∞	∞	∞	∞	-0.1	0.02	0.03
k__Bacteria	Actinobacteria	Propionibacteriaceae	Propionibacterium freudenreichii				0									-0.1	-0.1	-0.1							∞									-∞	-∞	-1.1			
k__Bacteria	Actinobacteria	Bifidobacteriaceae	Bifidobacterium adolescentis	-5.1	15.2	19.4	-0	-0	-0	-2.6	-10	-7.2				11.2	2.7	0.11	-6.9	-1	1.38	-1.8	0.6	0.68	-∞	-∞	-∞	-0.1	-0.7	-0.4				∞	∞	∞	-1.1	-0.1	0.07
k__Bacteria	Actinobacteria	Bifidobacteriaceae	Bifidobacterium animalis	-0.4	-0.4	4.54	0.01				0.01		0.01		1.9	-0	-0	-0	5.43	-0.1	-0.1	-∞	-∞	1.08	∞				∞		∞		∞	-0.7	-∞	-∞	1.93	-1.1	-∞
k__Bacteria	Actinobacteria	Bifidobacteriaceae	Bifidobacterium bifidum	-3.1	-3.3	-3.3	2.83					0.25				0	0.02	1.46	-6.1	-4.7	-1.8	-1.2	-2.3	-∞	∞					∞				∞	∞	∞	-0.3	-0.2	-0.1
k__Bacteria	Actinobacteria	Bifidobacteriaceae	Bifidobacterium breve			0									0.01				-0	-0.1	-0			∞									∞				-0.3	-0.4	-0.2
k__Bacteria	Actinobacteria	Bifidobacteriaceae	Bifidobacterium catenulatum	-0	-0	-0												0	-0	-0	-0	-∞	-1	-0												∞	-0.5	-0.4	-0.5
k__Bacteria	Actinobacteria	Bifidobacteriaceae	Bifidobacterium dentium													-0.2	0.34	-0.1		0														-0.5	0.41	-0.4		∞	∞
k__Bacteria	Actinobacteria	Bifidobacteriaceae	Bifidobacterium longum	0.65	0.18	0.34	0.59	-0	-0.2							1.9	-0	1.14	-0.1	-0.2	-6.8	0.2	0.07	0.12	0.39	-0.1	-0.4							0.45	-0	0.32	-0	-0	-0.2
k__Bacteria	Actinobacteria	Bifidobacteriaceae	Bifidobacterium pseudocatenulatum													-0.5	-0.4	0.14	7.54	4.83	2.8													-1.3	-0.9	0.11	0.72	0.57	0.41
k__Bacteria	Actinobacteria	Bifidobacteriaceae	Bifidobacterium unclassified										0.25	0.03	-0																1.1	0.38	-∞						
k__Bacteria	Actinobacteria	Coriobacteriaceae	Atopobium parvulum											0					-0	-0	-0											∞					-∞	-∞	-∞
k__Bacteria	Actinobacteria	Coriobacteriaceae	Atopobium rimae														0																		∞				
k__Bacteria	Actinobacteria	Coriobacteriaceae	Collinsella aerofaciens	0.03	5.23	0.39	-2.1	-3.1	-5.5	-1.8	-3.2	-2.3	-5.5	-3.3	-2.5	-0.1	0.46	-1.2	-1.1	-0.7	-0.7	0.01	0.54	0.07	-0.2	-0.3	-1.4	-0.1	-0.3	-0.2	-0.3	-0.2	-0.1	-0	0.05	-0.2	-0.2	-0.1	-0.1
k__Bacteria	Actinobacteria	Coriobacteriaceae	Collinsella intestinalis							1.25	-0.3	-0.3	0.29	0.46	0.01													0.67	-0.7	-0.6	∞	∞	∞						
k__Bacteria	Actinobacteria	Coriobacteriaceae	Collinsella stercoris			0				0	-0	-0	0	0					0.07	0.07	0.23			∞				0.21	-0.8	-∞	∞	∞					∞	∞	∞
k__Bacteria	Actinobacteria	Coriobacteriaceae	Eggerthella lenta	-0	-0	-0	-0.3	-0.3	-0.3	0.03	-0	0.16	-0.3	-0.3	-0.3	0.06	-0	-0	-0.6	-0.6	-0.6	-1.5	-0.9	-0.3	-1.5	-∞	-∞	0.11	-0.2	0.39	-0.5	-0.4	-0.4	1.46	-0.4	-0.9	-0.7	-0.8	-0.9
k__Bacteria	Actinobacteria	Coriobacteriaceae	Gordonibacter pamelaeae	0.03	0.03	-0	-0.1	-0.1	-0.1	0.16	0.08	0.12	0.17	0.2	0.43	0.36	-0	-0	-0	0.01	-0	0.16	0.14	-0.1	-1.1	-∞	-∞	0.48	0.31	0.39	∞	∞	∞	1.23	-0.2	-0	-∞	0.35	-0.2
k__Bacteria	Actinobacteria	Coriobacteriaceae	Olsenella uli	0		0													-0	-0	0	∞		∞													-∞	-∞	0.14
k__Bacteria	Actinobacteria	Coriobacteriaceae	Slackia exigua				-0	-0	-0		0.01														-∞	-∞	-∞		∞										
k__Bacteria	Actinobacteria	Coriobacteriaceae	Slackia unclassified	0	0.04	0.01	0.11	0.22	-0	0.05	-0	-0	0.02	0.04	0.03	-0.1	-0.1	-0.1	-0	-0	-0	0.21	0.87	0.47	0.59	0.83	-0.4	0.21	-0.1	-0.1	0.72	0.87	0.78	-0.3	-0.6	-0.3	-0.3	-0.2	-0.2
k__Bacteria	Bacteroidetes	Bacteroidaceae	Bacteroides caccae	-0.2	-0.2	-0.2	1.49	0.1	0.55							0.33	-0.5	-0.4	0.09	0.43	0.36	-∞	-1	-0.9	∞	∞	∞							0.14	-0.4	-0.3	∞	∞	∞
k__Bacteria	Bacteroidetes	Bacteroidaceae	Bacteroides cellulosilyticus				-0.5	-0.5	-0.5	0.01	0.04	0.04				0.14	0.06	0.03	0.1	0.18	2.26				-2.2	-∞	-∞	∞	∞	∞				∞	∞	∞	∞	∞	∞
k__Bacteria	Bacteroidetes	Bacteroidaceae	Bacteroides coprophilus														0	0																	∞	∞			
k__Bacteria	Bacteroidetes	Bacteroidaceae	Bacteroides dorei	-0.1	-0.2	-0.2				0.2	0.26	0.17	0.27	0.16	0.25	0.04	0.07	0.1	0	0	0.05	-0.4	-1.2	-1.1				0.8	0.9	0.75	1.79	1.56	1.76	0.57	0.79	0.93	∞	∞	∞
k__Bacteria	Bacteroidetes	Bacteroidaceae	Bacteroides eggerthii				0						0	0	-0	-0	0	0							∞									0.02	0.04	-0.6			
k__Bacteria	Bacteroidetes	Bacteroidaceae	Bacteroides finegoldii	0	-0	-0										-0.4	-0.3	-0.3	1.16	2.21	2.18	0.64	-∞	-∞										-1.3	-0.8	-0.7	∞	∞	∞
k__Bacteria	Bacteroidetes	Bacteroidaceae	Bacteroides fragilis				0.33	-0.2	-0.3	0.06	-0	-0.1	0.03	0.13	0.04	0.01			-0	-0	-0				0.31	-0.4	-1.3	0.11	-0	-0.4	0.31	0.76	0.4	∞			-0.3	-0.4	-0.5
k__Bacteria	Bacteroidetes	Bacteroidaceae	Bacteroides helcogenes																																				
k__Bacteria	Bacteroidetes	Bacteroidaceae	Bacteroides intestinalis							0.09	0.38	0.36				2.77	-1.1	-1.2	0.02	0.18	1.3							1.03	1.6	1.58				0.52	-1.2	-4.1	∞	∞	∞
k__Bacteria	Bacteroidetes	Bacteroidaceae	Bacteroides ovatus	-0.2	-0.1	-0.2	-0.1	-0.2	-0.2	0.01	0.05	0.01	-0.7	-0.7	0.12	0.1	-0	0.04	-1.7	-2.2	0.44	-0.4	-0.2	-0.8	-0.5	-∞	-∞	0.15	0.45	0.11	-1.1	-1.4	0.07	0.86	-0.6	0.53	-0.4	-0.7	0.06
k__Bacteria	Bacteroidetes	Bacteroidaceae	Bacteroides pectinophilus															0																		∞			
k__Bacteria	Bacteroidetes	Bacteroidaceae	Bacteroides plebeius	0.24	2.03	1.49				-0.8	-0.1	-0.1	0.74	0.94	0.06	-0.1	0.16	0.14				∞	∞	∞				-0.4	-0	-0	∞	∞	∞	-0.5	0.35	0.32			
k__Bacteria	Bacteroidetes	Bacteroidaceae	Bacteroides salanitronis				-0	-0	-0							0	0	0							-0.7	-∞	-∞							∞	∞				
k__Bacteria	Bacteroidetes	Bacteroidaceae	Bacteroides stercoris				0.38	0.12	0.63				-0.8	-1.5	-5.8	0.08	0.12	0.25	-3.2	-2.3	0.96				∞	∞	∞				-0	-0.1	-0.5	0.12	0.17	0.3	-0.2	-0.1	0.04
k__Bacteria	Bacteroidetes	Bacteroidaceae	Bacteroides thetaiotaomicron	0.1	-0	-0	-0	-0	-0				-0.2	-0.4	-0.3	-0.3	-0.3	-0.3	-0	-0	-0	0.4	-0.1	-0.3	-∞	-∞	-∞				-0.3	-0.7	-0.5	-0.5	-0.6	-0.4	-1.3	-0.5	-0.2
k__Bacteria	Bacteroidetes	Bacteroidaceae	Bacteroides unclassified	-0.4	-4.7	-4	-3.5	-7.7	-7.7	6.52	14.7	7.88	0.99	0.61	-12	-3.2	-2.6	-1.7	0.53	-2.6	-1.5	-0	-2.1	-0.8	-0.3	-∞	-∞	0.2	0.37	0.23	0.02	0.01	-0.3	-∞	-0.8	-0.3	0.08	-∞	-0.4
k__Bacteria	Bacteroidetes	Bacteroidaceae	Bacteroides uniformis			0.04	-0.1	-0.2	-0.2	0.17	0.57	0.02	0.22	0.31	0.35	0.17	0.01	0.16	0.07	0.43	0.53			∞	-0.5	-∞	-1.5	1.47	1.98	0.7	0.15	0.2	0.22	1.2	0.35	1.19	0.58	1.26	1.35
k__Bacteria	Bacteroidetes	Bacteroidaceae	Bacteroides vulgatus	-0.4	-1.6	-1.6	-4.1	0.46	-0.3	1.71	2.16	0.75	-0.5	-1.4	-2.3	0.96	0.8	1.53	-3.7	0.66	1.16	-0.1	-1.6	-1.4	-0.4	0.03	-0	0.47	0.54	0.27	-0.1	-0.2	-0.4	0.4	0.36	0.54	-0.2	0.03	0.05
k__Bacteria	Bacteroidetes	Bacteroidaceae	Bacteroides xylanisolvens	-0.9	-1.3	-1.3	0.09	-0	0.32	0.41	0.89	1.06	0.64	0.28	-0.1	-0	-0.1	-0	-0	0.02	0.39	-0.5	-1.3	-1.4	0.76	-0.2	1.27	0.31	0.51	0.57	0.35	0.19	-0.1	-0.1	-0.2	-0.1	-0	0.03	0.39
k__Bacteria	Bacteroidetes	Porphyromonadaceae	Odoribacter splanchnicus	0.03	0.05	0.07	-0.3	-0.4	-0.4	0.31	0.63	0.35	0.49	0.07	-0.1	1.35	-0.1	0.05	-1.1	-0.9	-0.5	∞	∞	∞	-0.7	-∞	-1.9	0.33	0.52	0.36	0.35	0.07	-0.2	0.48	-0.1	0.03	-2.2	-0.8	-0.2
k__Bacteria	Bacteroidetes	Porphyromonadaceae	Parabacteroides distasonis			0							-0	-0	-0	-0	-0.1	-0.1	0.11	0.01	0			∞							-1.8	-∞	-2	-0	-0.7	-0.6	0.75	0.15	0.05
k__Bacteria	Bacteroidetes	Porphyromonadaceae	Parabacteroides johnsonii													0	0	0																∞	∞	∞			
k__Bacteria	Bacteroidetes	Porphyromonadaceae	Parabacteroides merdae	0		0	0		0.04	0.12	0.26	0.09	0.72	1.34	0.65	0.03	-0.2	0.5	-0.2	0.74	0.75	∞		∞	∞		∞	0.25	0.43	0.2	∞	∞	∞	0.03	-0.3	0.33	-0.1	0.38	0.38
k__Bacteria	Bacteroidetes	Porphyromonadaceae	Parabacteroides unclassified	0		0				-0	0.03	-0	0.08	0.23		0.01		0.06	-0.1	0.35	0.27	∞		∞				-0.1	0.09	-0.1	∞	∞		∞		∞	-0.7	0.5	0.43
k__Bacteria	Bacteroidetes	Porphyromonadaceae	Porphyromonas asaccharolytica				0.03	-0	-0							1.13	-0	-0							0.56	-0.6	-∞							1.61	-∞	-∞			
k__Bacteria	Bacteroidetes	Porphyromonadaceae	Porphyromonas uenonis													0.04	-0	-0																0.31	-∞	-∞			
k__Bacteria	Bacteroidetes	Prevotellaceae	Prevotella buccae																																				
k__Bacteria	Bacteroidetes	Prevotellaceae	Prevotella buccalis			0																		∞															
k__Bacteria	Bacteroidetes	Prevotellaceae	Prevotella copri	0.2	2.89	0.21	20.9	22.3	75.6							-12	24.8	20.1				∞	∞	∞	1.55	1.58	2.1							-0.7	0.42	0.37			
k__Bacteria	Bacteroidetes	Prevotellaceae	Prevotella disiens																																				
k__Bacteria	Bacteroidetes	Prevotellaceae	Prevotella oris						0.02																		∞												
k__Bacteria	Bacteroidetes	Rikenellaceae	Alistipes putredinis	0.01		0.01	0.11			2.01	3.61	3.56	-0.5	-0.9	-1.5	-0.2	-7.8	-8.3	0.14	2.47	6.47	∞		∞	∞			0.53	0.72	0.71	-0.1	-0.1	-0.2	-0	-0.4	-0.4	0.39	1.43	1.84
k__Bacteria	Bacteroidetes	Rikenellaceae	Alistipes shahii	0.01	0.04	0.05	-0.3	-0.6	-0.6	0.55	1.52	0.95	0.21	0.24	0.32	9.12	-0.3	-0.4	-0	0.29	0.47	∞	∞	∞	-0.4	-∞	-1.8	∞	∞	∞	0.23	0.26	0.32	0.94	-0.1	-0.2	-0.2	0.78	0.96
k__Bacteria	Bacteroidetes	Rikenellaceae	Alistipes unclassified	0			0		0.01										-0.3	-0.3	-0.3	∞			∞		∞										-∞	-∞	-∞
k__Bacteria	Bacteroidetes	Flavobacteriaceae	Cellulophaga unclassified																																				
k__Bacteria	Bacteroidetes	Flavobacteriaceae	Flavobacterium unclassified							0						-0	-0	-0										∞						-0.1	-0.2	-0.4			
k__Bacteria	Firmicutes	Bacillales uncl	Gemella haemolysans																		0																		∞
k__Bacteria	Firmicutes	Bacillales uncl	Gemella moribillum				-0	-0	-0	-0.1	-0.1	0.38				-1.2	-1.4	-1.7	-0	-0	-0				-∞	-∞	-∞	-0.6	-0.3	0.46				-0.5	-0.7	-2.2	-∞	-∞	-∞
k__Bacteria	Firmicutes	Bacillales uncl	Gemella unclassified				-0	-0	-0	-0	-0	-0									0				-0.9	-0.1	-∞	-0.5	-0.7	-∞						∞			
k__Bacteria	Firmicutes	Aerococcaceae	Abiotrophia defectiva					0																		∞													
k__Bacteria	Firmicutes	Carnobacteriaceae	Granulicatella adiacens	0	-0	0	0	0.02	-0	-0.1	-0.1	-0.1	0	0					-0	-0	0.01	0.45	-∞	0.7	0.4	0.9	-∞	-1.4	-0.7	-0.9	∞	∞					-∞	-∞	0.6
k__Bacteria	Firmicutes	Carnobacteriaceae	Granulicatella unclassified	-0	-0	-0	0.02	0.06	-0	-0	-0	-0	0	-0	-0			0	0	-0	0.03	-0.5	-∞	-0.6	0.42	0.89	-∞	-0.5	-0.8	-1.3	0.26	-∞	-∞			∞	0.1	-∞	1.21
k__Bacteria	Firmicutes	Enterococcaceae	Enterococcus faecalis			0													0					∞													∞		
k__Bacteria	Firmicutes	Enterococcaceae	Enterococcus faecium																																				
k__Bacteria	Firmicutes	Enterococcaceae	Enterococcus unclassified	-0	-0	-0	0	-0	-0	0.01	0	-0	-0	-0	-0		0	0	-0	-0	-0	-0.6	-0.3	-1.1	0.03	-0.1	-∞	0.24	0.01	-0.3	-0.6	-0.1	-0.2		∞	∞	-1.2	-0	-0.1
k__Bacteria	Firmicutes	Lactobacillaceae	Lactobacillus acidophilus	-0	-0	0.35									0.04				1.03			-∞	-∞	1.53									∞				∞		
k__Bacteria	Firmicutes	Lactobacillaceae	Lactobacillus brevis			0.01		0.02																∞		∞													
k__Bacteria	Firmicutes	Lactobacillaceae	Lactobacillus casei					0.06								0.01		0								∞								∞		∞			
k__Bacteria	Firmicutes	Lactobacillaceae	Lactobacillus delbrueckii	-0	-0	-0	0.66	1.3														-∞	-∞	-∞	∞	∞													
k__Bacteria	Firmicutes	Lactobacillaceae	Lactobacillus fermentum														0																		∞				
k__Bacteria	Firmicutes	Lactobacillaceae	Lactobacillus gasseri				2.81	3.65																	∞	∞													
k__Bacteria	Firmicutes	Lactobacillaceae	Lactobacillus iners									0.01																		∞									
k__Bacteria	Firmicutes	Lactobacillaceae	Lactobacillus paracasei					0.18										0								∞										∞			
k__Bacteria	Firmicutes	Lactobacillaceae	Lactobacillus plantarum			0.06									0.07		0							∞									∞		∞				
k__Bacteria	Firmicutes	Lactobacillaceae	Lactobacillus rhamnosus	-0	-0	0										0.02	0.05		-0	-0	-0	-∞	-∞	0.1										∞	∞		-∞	-∞	-∞
k__Bacteria	Firmicutes	Lactobacillaceae	Lactobacillus ruminis	2.78	4.2	3.51																∞	∞	∞															
k__Bacteria	Firmicutes	Lactobacillaceae	Lactobacillus sakei															0	-0.1	-0.1	-0.1															∞	-∞	-∞	-∞
k__Bacteria	Firmicutes	Lactobacillaceae	Pediococcus acidilactici																																				
k__Bacteria	Firmicutes	Lactobacillaceae	Pediococcus pentosaceus																																				
k__Bacteria	Firmicutes	Leuconostocaceae	Leuconostoc gasicomitatum															0	-0	-0	-0															∞	-∞	-∞	-∞
k__Bacteria	Firmicutes	Leuconostocaceae	Leuconostoc kimchii																-0.1	-0.1	-0.1																-∞	-∞	-∞
k__Bacteria	Firmicutes	Leuconostocaceae	Leuconostoc mesenteroides	-0	-0	-0													-0	-0	-0	-∞	-∞	-∞													-∞	-∞	-∞
k__Bacteria	Firmicutes	Leuconostocaceae	Leuconostoc unclassified																-0	-0	-0																-∞	-∞	-∞
k__Bacteria	Firmicutes	Streptococcaceae	Lactococcus lactis	0.02	0.32		-1.1	0.22	-1.1							0.08	-0	0.02	-0.1	-0.1	-0.1	∞	∞		-∞	0.08	-∞							1.47	-0.1	0.81	-∞	-0.4	-0.3
k__Bacteria	Firmicutes	Streptococcaceae	Streptococcus agalactiae																																				
k__Bacteria	Firmicutes	Streptococcaceae	Streptococcus anginosus			0	-0.1	-0	-0.2							0.13	0.04	0.01	-0	-0	-0			∞	-0.9	-0.2	-∞							∞	∞	∞	-∞	-∞	-0.7
k__Bacteria	Firmicutes	Streptococcaceae	Streptococcus australis	0.09	0.05	0.03	-0.2	-0	-0.3	-0.1	-0.1	-0.1	0.01	0	0		0	0	-0	-0.1	-0	1.02	0.8	0.61	-0.5	-0	-2	-0.4	-0.6	-1.5	∞	∞	∞		∞	∞	-0.4	-1.4	-0.4
k__Bacteria	Firmicutes	Streptococcaceae	Streptococcus cristatus																																				
k__Bacteria	Firmicutes	Streptococcaceae	Streptococcus equinus				0.01	0.06																	∞	∞													
k__Bacteria	Firmicutes	Streptococcaceae	Streptococcus gordonii																																				
k__Bacteria	Firmicutes	Streptococcaceae	Streptococcus infantarius				0.28	1.86	0.01																∞	∞	∞												
k__Bacteria	Firmicutes	Streptococcaceae	Streptococcus infantis	0.01		0	-0.1	0.04	-0.1	-0	-0	-0	0						-0	-0	-0	∞		∞	-∞	0.22	-∞	-∞	-∞	-∞	∞						-∞	-∞	-0.2
k__Bacteria	Firmicutes	Streptococcaceae	Streptococcus mitis	-0	-0	-0	-0	0.16	-0	0.01	-0	-0	-0	0.01	-0		0		-0	-0	0.01	-∞	-∞	-0.5	-0.2	0.72	-0.5	0.62	-∞	-∞	-∞	0.43	-∞		∞		-∞	-0.4	0.12
k__Bacteria	Firmicutes	Streptococcaceae	Streptococcus mutans														0	0																	∞	∞			
k__Bacteria	Firmicutes	Streptococcaceae	Streptococcus oralis																-0	-0	-0																-∞	-∞	-∞
k__Bacteria	Firmicutes	Streptococcaceae	Streptococcus parasanguinis	0.07	0.03	0.01	-0.7	-0.6	-0.7	-0.1	-0.1	-0.1	0.03	-0	0.11		0.08	0.02	-0	-0	0.35	1.48	1.16	0.79	-1.4	-1.1	-2.6	-0.5	-0.8	-2.5	0.14	-0	0.39		∞	∞	-0.2	-0.1	0.72
k__Bacteria	Firmicutes	Streptococcaceae	Streptococcus pneumoniae																																				
k__Bacteria	Firmicutes	Streptococcaceae	Streptococcus salivarius	0.18	0.06	0.12	-1	-1	-1	-0.1	-0.1	-0.1	-0	0.08	0.09	0	0.05	0.02	-0.5	0.37	0.87	0.8	0.42	0.64	-∞	-3.1	-1.8	-0.8	-0.4	-1.4	-0.2	0.3	0.35	∞	∞	∞	-0.7	0.19	0.36
k__Bacteria	Firmicutes	Streptococcaceae	Streptococcus sanguinis	0.02		0.01	-0.1	-0.1	-0.1						0		0	0	-0	-0	0.02	∞		∞	-∞	-1.7	-∞						∞		∞	∞	-∞	-∞	0.55
k__Bacteria	Firmicutes	Streptococcaceae	Streptococcus thermophilus	-0.7	-0.1	1.43	19.1	24.4	-0.6		0			0	9.1	-0	-0	0.59	0.59	1.23	0.6	-1.6	-0	0.47	1.5	1.6	-1.4		∞			∞	∞	-∞	-1.6	1.13	0.44	0.67	0.45
k__Bacteria	Firmicutes	Streptococcaceae	Streptococcus vestibularis	0.03	0.02	0.02	-0	-0	-0				0	0.02	0			0.01	-0	0	0.06	∞	∞	∞	-∞	-∞	-∞				∞	∞	∞			∞	-∞	0.09	0.76
k__Bacteria	Firmicutes	Clostridiaceae	Alkaliphilus unclassified				0.01			0.01	0	0	0.01		0	0.01	-0	0.02	-0	-0	-0				∞			∞	∞	∞	∞		∞	0.33	-0.2	0.71	-∞	-∞	-∞
k__Bacteria	Firmicutes	Clostridiaceae	Clostridium asparagiforme				-0	-0	-0	0.01	0.01	-0	-0	-0	-0	0.02	-0	0							-∞	-∞	-∞	0.73	0.65	-0.6	-0.4	-0.5	-0.7	1.15	-1	0.18			
k__Bacteria	Firmicutes	Clostridiaceae	Clostridium bartlettii		0.01	0.03	-0	0.01	-0	0.03			0.1		0.03	-0	-0	0.02	0.01	0.12	-0.1		∞	∞	-1.2	0.47	-∞	∞			∞		∞	-0.2	-0.8	0.41	0.02	0.29	-0.5
k__Bacteria	Firmicutes	Clostridiaceae	Clostridium bolteae							0.03	0	-0	0.01	-0	0	0	-0	-0	-0	-0	-0							1.07	0.38	-0	0.48	-0.2	0.4	0.37	-∞	-∞	-∞	-∞	-∞
k__Bacteria	Firmicutes	Clostridiaceae	Clostridium botulinum								0.01					-0	-0	-0											∞					-0.2	-∞	-∞			
k__Bacteria	Firmicutes	Clostridiaceae	Clostridium cf	-0	-0	-0	-0	-0	-0	0.01	-0	0.01	0.02	-0	-0	-0	-0	-0	-0.1	-0.1	-0.2	-0.1	-0.1	-0.3	-∞	-∞	-∞	0.19	-0.1	0.35	0.5	-0.6	-0.2	-0.2	-0.4	-0.3	-0.2	-0.5	-0.9
k__Bacteria	Firmicutes	Clostridiaceae	Clostridium hathewayi				-0	-0	-0	0.01	0	0	0	0.01	0.01	-0	-0	-0	-0	-0	-0				-∞	-∞	-∞	0.62	0.09	0.09	∞	∞	∞	-0.6	-∞	-∞	-∞	-∞	-∞
k__Bacteria	Firmicutes	Clostridiaceae	Clostridium hylemonae													0																		∞					
k__Bacteria	Firmicutes	Clostridiaceae	Clostridium leptum							-0	0.05	-0	0.14	0.03	0.05	0.07	-0	0.14										-0.5	0.37	-0.3	∞	∞	∞	0.15	-0.1	0.27			
k__Bacteria	Firmicutes	Clostridiaceae	Clostridium methylpentosum							0	0	0.01				-0	-0	-0										∞	∞	∞				-0	-∞	-∞			
k__Bacteria	Firmicutes	Clostridiaceae	Clostridium nexile			0	0.64	2.2	0.25	-0	0	-0	-0	-0	-0	0	0	0	-0	0	0			∞	1.43	1.96	1.05	-0.1	0.11	-0.4	-0.2	-0.2	-0.1	0.25	0.1	0.29	-∞	0.26	0.03
k__Bacteria	Firmicutes	Clostridiaceae	Clostridium saccharolyticum				-0	-0	-0	0	0		0				0	0							-∞	-∞	-∞	∞	∞		∞				∞	∞			
k__Bacteria	Firmicutes	Clostridiaceae	Clostridium scindens			0	-0	-0	-0	0	0.01	0.01	-0	-0	-0	0.04	####	0	####	-0	-0			∞	-1.4	-∞	-∞	0.26	0.43	0.34	-0.2	-0.2	-0.2	1.29	0.01	0.15	-0	-0.2	-∞
k__Bacteria	Firmicutes	Clostridiaceae	Clostridium symbiosum				-0	0.02	-0	-0	-0	0	0		0				-0	-0	-0				-1.2	0.3	-∞	-∞	-∞	0.2	∞		∞				-∞	-∞	-∞
k__Bacteria	Firmicutes	Clostridiaceae	Pseudoflavonifractor capillosus	0.02	-0	-0	-0	-0	-0	0.04	0.01	0.01	-0	0.01	0.01	-0	-0	0.01	-0.1	-0.1	-0.1	0.62	-0.3	-0.1	-0.3	-∞	-0.4	0.47	0.22	0.13	-0	0.19	0.12	-0.3	-0.2	0.14	-0.6	-0.5	-0.7
k__Bacteria	Firmicutes	Clostridiales Family XI Inc	Anaerococcus prevotii																																				
k__Bacteria	Firmicutes	Clostridiales Family XI Inc	Anaerococcus unclassified	0		0	-0	-0	-0	-0.1	0.05	6.41			0.06	-0.1	-0.1	-0.1	-0	-0	-0	∞		∞	-∞	-∞	-∞	-0.3	0.12	1.66			∞	-0.5	-1.1	-2	-∞	-∞	-∞
k__Bacteria	Firmicutes	Clostridiales Family XI Inc	Anaerococcus vaginalis							-0	0.02	3.43				-0.1	-0.1	-0.1										-0.4	0.13	1.83				-∞	-∞	-∞			
k__Bacteria	Firmicutes	Clostridiales Family XI Inc	Finegoldia magna												0.05																		∞						
k__Bacteria	Firmicutes	Clostridiales Family XI Inc	Parvimonas micros				0.01	0.02	-0	-0.1	0.19	4.29				-0.1	-0.2	-0.2							0.25	0.38	-∞	-0.7	0.36	1.48				-0.2	-0.8	-2.4			
k__Bacteria	Firmicutes	Clostridiales Family XI Inc	Peptoniphilus duerdenii												0.01																		∞						
k__Bacteria	Firmicutes	Clostridiales Family XI Inc	Peptoniphilus harei							-0.1	-0	4.9			0.02	-2.1	-2.1	-2.1										-0.6	-0.1	1.56			∞	-∞	-∞	-∞			
k__Bacteria	Firmicutes	Clostridiales Family XI Inc	Peptoniphilus lacrimalis																																				
k__Bacteria	Firmicutes	Clostridiales Family XI Inc	Peptoniphilus unclassified	0.01	-0	-0	-0	0.02	-0.1	0.03	0.06	0.47	-0	-0.1	0	0.46	0.09	0.03	-0	-0	-0	0.17	-0.1	-0	-0.1	0.07	-1	0.48	0.68	1.51	-0.3	-0.3	0.01	∞	∞	∞	-0.2	-0.2	-0.4
k__Bacteria	Firmicutes	Clostridiales uncl	Blautia hansenii				-0	-0	-0	0	0	-0	-0	-0	-0	0	-0	-0	-0.3	-0.3	1.13				-0.3	-∞	-∞	0.45	0.33	-∞	-0.5	-0.4	-0.5	0.13	-0.1	-0.2	-0.7	-0.7	0.66
k__Bacteria	Firmicutes	Clostridiales uncl	Blautia hydrogenotrophica	-0.5	-0.8	-0.7	-0	-0	-0	0.19	-0.1	-0.2	-0.5	-0.5	-0.4	0.01	-0	0	-1.3	-1.3	-1.3	-0.3	-0.5	-0.4	-∞	-∞	-∞	0.24	-0.4	-1.1	-1	-1.1	-0.8	0.43	-0.3	0.03	-0.5	-0.5	-0.5
k__Bacteria	Firmicutes	Clostridiales uncl	Blautia unclassified	0.21	0.43	-0	-2.8	-2.3	-3.7	-1.2	-0.3	-2.6	-0.4	0.02	-0.3	-0.3	-0.4	-0.3	0.45	0.38	0.12	0.26	0.42	-0	-0.5	-0.3	-0.9	-0.2	-0	-0.7	-0.1	0.01	-0.1	-0.2	-0.2	-0.2	∞	∞	∞
k__Bacteria	Firmicutes	Eubacteriaceae	Anaerofustis stercorihominis																																				
k__Bacteria	Firmicutes	Eubacteriaceae	Eubacterium cellulosolvens														0	0																	∞	∞			
k__Bacteria	Firmicutes	Eubacteriaceae	Eubacterium eligens	-2.7	1.83	0.62	4.67	-0	-0	-5.7	-5.7	-8	-2.1	-3.6	-2.8	-2.1	-1.2	-0.6	0.57	-1.3	-1.6	-2	0.22	0.09	2.44	-∞	-∞	-0.3	-0.3	-0.5	-0.1	-0.3	-0.2	-0.4	-0.2	-0.1	0.13	-0.6	-1.4
k__Bacteria	Firmicutes	Eubacteriaceae	Eubacterium hallii	-0.9	0.88	0.44	-1.1	-1.4	-1.5	0.12	-0.6	-0.7	0.97	0.37	2.08	0.43	-0	0.02	-0.1	-0	-0.2	-0.4	0.2	0.11	-0.5	-0.9	-1.2	0.03	-0.2	-0.2	0.18	0.08	0.32	0.57	-0.2	0.05	-0.2	-0	-0.3
k__Bacteria	Firmicutes	Eubacteriaceae	Eubacterium limosum													-0	-0	-0	-0	-0	-0													-0.2	-0.9	-0.8	-∞	-∞	-∞
k__Bacteria	Firmicutes	Eubacteriaceae	Eubacterium rectale	2.61	-31	-25	0.16	-0	-0	-0.2	0.32	-2.1	2.94	-3.6	9.38	0.18	7.41	8.89	3.31	6.32	0.74	0.03	-0.6	-0.4	0.67	-∞	-∞	-0	0.02	-0.2	0.18	-0.4	0.42	0.06	0.86	0.93	0.43	0.62	0.14
k__Bacteria	Firmicutes	Eubacteriaceae	Eubacterium siraeum												0	-10	-12	-13															∞	-0.6	-0.9	-1.4			
k__Bacteria	Firmicutes	Eubacteriaceae	Eubacterium ventriosum	2.38	0.23	7.96	-0	-0.1	-0.1	-0	0.01	-0	0.06	1.7	0.35	-0.2	0.35	0.19	1.93	1.86	-0	1.06	0.31	1.56	-0.6	-∞	-∞	-0.1	0.04	-0.4	0.18	1.16	0.58	-0.5	0.4	0.25	0.85	0.84	-0
k__Bacteria	Firmicutes	Lachnospiraceae	Anaerostipes caccae													0																		∞					
k__Bacteria	Firmicutes	Lachnospiraceae	Butyrivibrio crossotus															0																		∞			
k__Bacteria	Firmicutes	Lachnospiraceae	Butyrivibrio unclassified	-0	0.04	-0	-0	-0	-0.1	-0	-0.1	-0.1	0.03	0.02	-0	0.01	0.01	-0	-0	-0	-0	-0.1	0.22	-0.4	-0.4	-0.3	-∞	-0	-0.2	-0.5	0.13	0.11	-0.1	0.21	0.22	-0.3	-0.2	-0.2	-0.3
k__Bacteria	Firmicutes	Lachnospiraceae	Coprococcus catus	-0.1	0.03	-0.3	-0.3	-0.3	-1.8	0.14	-0.1	-0.3	0.05	0.04	0.36	0.08	-0.3	-0.3	0.43	0.19	-0.4	-0.1	0.03	-0.3	-0.1	-0.1	-0.9	0.12	-0.2	-0.6	0.06	0.06	0.36	0.04	-0.2	-0.2	0.23	0.12	-0.4
k__Bacteria	Firmicutes	Lachnospiraceae	Coprococcus comes	-0.1	0.4	-1.1	-5.6	-7	-7.1	-0.9	-1.1	-1.4	0.64	1.47	2.74	0.49	0.87	0.54	0.26	0.92	0.23	-0	0.06	-0.3	-0.5	-1	-1.1	-0.3	-0.4	-0.7	0.1	0.19	0.31	0.76	0.97	0.79	0.12	0.33	0.11
k__Bacteria	Firmicutes	Lachnospiraceae	Coprococcus eutactus																																				
k__Bacteria	Firmicutes	Lachnospiraceae	Dorea formicigenerans	0.83	1.31	0.74	-0.2	1.07	-0.9	-0.1	0.07	0.14	-0.5	-0.1	0.83	0.4	0.51	0.39	-0.6	-2	-0.7	0.22	0.31	0.2	-0.1	0.26	-0.5	-0	0.02	0.04	-0.1	-0	0.14	0.32	0.37	0.31	-0.1	-0.4	-0.1
k__Bacteria	Firmicutes	Lachnospiraceae	Dorea longicatena	1.01	3.66	-0.9	-2.7	-2.5	-3.6	0.37	-0.2	0.75	-0.8	0.35	1.57	-0.2	1.32	0.82	-0.4	0.23	1.9	0.09	0.25	-0.1	-0.5	-0.4	-1	0.07	-0	0.13	-0.2	0.06	0.22	-0	0.23	0.16	-0.2	0.08	0.42
k__Bacteria	Firmicutes	Lachnospiraceae	Marvinbryantia formatexigens			0	-0	-0	-0	0	0	-0	0	0	0	-0.3	-0.3	-0.3						∞	-∞	-∞	-∞	0.36	0.2	-1.1	∞	∞	∞	-0.5	-0.7	-0.9			
k__Bacteria	Firmicutes	Lachnospiraceae	Oribacterium sinus	0																		-∞																	
k__Bacteria	Firmicutes	Lachnospiraceae	Roseburia intestinalis	-0.7	2.35	0.72	-0	-0	-0	1.32	2.86	0	0.15	-0.3	0.82	-0.2	0.33	0.72	8.17	2.15	0.37	-0.8	0.6	0.28	-0.3	-∞	-∞	2.41	2.74	0.02	0.05	-0.1	0.21	-1	0.42	0.66	1.3	0.78	0.27
k__Bacteria	Firmicutes	Lachnospiraceae	Roseburia inulinivorans	-0.3	-1.4	-1.4	-1.5	-2.5	-2	-0.6	0.12	-1.1	0.01	1.29	-0.1	-0.6	-0.5	-0.2	2.72	1.84	0.8	-0.1	-0.5	-0.5	-0.4	-∞	-0.7	-0.3	0.04	-2.1	0.01	0.4	-0.1	-1	-0.5	-0.2	1.35	1.19	0.86
k__Bacteria	Firmicutes	Lachnospiraceae	Roseburia unclassified					0.02				0.02														∞				∞									
k__Bacteria	Firmicutes	Peptostreptococcaceae	Peptostreptococcus anaerobius												0.03	0.62	0.01	0	-0.1	-0.1	-0.1												∞	∞	∞	∞	-∞	-∞	-∞
k__Bacteria	Firmicutes	Peptostreptococcaceae	Peptostreptococcus stomatis				0.26	0.23	-0	-0.1	0.01	0.69				3.41	0.36	-0.5			0.05				1.04	1.01	-∞	-0.7	0.03	0.7				0.89	0.23	-1.8			∞
k__Bacteria	Firmicutes	Peptostreptococcaceae	Peptostreptococcus unclassified										0						-0	-0	-0										∞						-∞	-∞	-∞
k__Bacteria	Firmicutes	Ruminococcaceae	Anaerotruncus colihominis				0.02		0	0	0	0	0	0	0.01	0.04	-0	-0							∞		∞	1.03	0.99	0.95	∞	∞	∞	1.25	-0.3	-0			
k__Bacteria	Firmicutes	Ruminococcaceae	Faecalibacterium cf	0.94	2.79	3	-0.9	-0.9	-1.2	-0	-0.4	-1.4	-2.4	-1.4	-3	0.18	0.52	0.49	-2.4	-3	-4.4	0.19	0.43	0.45	-0.3	-0.3	-0.5	-0	-0.1	-0.2	-0.5	-0.2	-0.9	0.1	0.25	0.23	-0.3	-0.5	-1.3
k__Bacteria	Firmicutes	Ruminococcaceae	Faecalibacterium prausnitzii	10.1	1.51	-0.4	-13	-15	-16	-6.1	-3	-6.3	5.36	7.23	-1	2.08	2.6	2.28	-2.5	-4	-8	0.27	0.05	-0	-0.3	-0.4	-0.5	-0.2	-0.1	-0.2	0.26	0.32	-0.1	0.26	0.3	0.28	-0.1	-0.3	-1.1
k__Bacteria	Firmicutes	Ruminococcaceae	Faecalibacterium unclassified	-0.3	-0.2	0.14	-2.4	-2.3	-3.4	-0.9	-2.7	-4.8	0.66	1.06	-0.2	-0.1	0.13	-0	-1.2	-1.3	-2.4	-∞	-0.6	0.18	-0.3	-0.3	-0.6	-0.1	-0.2	-0.4	0.35	0.48	-0.2	-∞	0.46	-0.3	-0.3	-0.3	-∞
k__Bacteria	Firmicutes	Ruminococcaceae	Ruminococcus albus	0	-0	-0				0.02	-0	-0	0.01	0	0.01	-0	####	0	-0	-0	-0	0.22	-0.3	-0.4				0.43	-0.2	-0.2	∞	∞	∞	-0.5	-0	0.2	-0.4	-0.6	-∞
k__Bacteria	Firmicutes	Ruminococcaceae	Ruminococcus bromii				-0	-0	-0							-4.7	-1.1	-2.5							-∞	-∞	-∞							-2.2	-0.1	-0.3			
k__Bacteria	Firmicutes	Ruminococcaceae	Ruminococcus gnavus			0	-0.1	3.54	-0.1	0.02	0	-0	-0.2	-0.2	-0.1	0	-0	-0	0.03	0.03	0.08			∞	-0.7	1.37	-0.5	1.1	0.54	-∞	-0.7	-0.9	-0.3	0.07	-0.5	-0.3	0.43	0.43	0.69
k__Bacteria	Firmicutes	Ruminococcaceae	Ruminococcus lactaris	0	0	-0	-0	-0	-0	2.08	0.02	0	-1.5	-1.8	-0.8	-0	1.19	0.75	-0	0	-0	0.21	0.15	-0	-0.1	-0.2	-1	2.55	0.66	0.05	-0.3	-0.4	-0.1	-0.5	1.36	1.16	-0	0.15	-∞
k__Bacteria	Firmicutes	Ruminococcaceae	Ruminococcus obeum	-5.8	-1.2	-5.6	-1.1	-2.1	-2.5	2.61	0.7	-1.1	-1.5	1.47	0.12	-4.4	-4.7	-5.5	0	0.53	0.02	-0.8	-0.1	-0.7	-0.2	-0.6	-1	0.34	0.12	-0.3	-0.3	0.17	0.02	-0.5	-0.6	-0.9	0	0.38	0.02
k__Bacteria	Firmicutes	Ruminococcaceae	Ruminococcus torques	-0	-0.1	-0.1	-2.2	-1.7	-2.6	1.89	-0.3	-0.5	0.46	-0.5	1.01	1.21	-0.9	-0.3	0.03	0.03	0.59	-0	-0.3	-0.4	-0.6	-0.4	-0.9	0.51	-0.2	-0.4	0.06	-0.1	0.13	0.23	-0.3	-0.1	0.15	0.12	0.89
k__Bacteria	Firmicutes	Ruminococcaceae	Subdoligranulum variabile	0.01	-0	-0	0.03	-0	-0	0.05	-0	-0	0.01	0.05	0.05	-0	-0	0.09	-0.1	-0	-0.1	0.28	-0.1	-0.2	0.24	-0.9	-0.7	0.26	-0.2	-0.1	0.1	0.33	0.35	-0.3	-0.2	0.28	-0.6	-0.5	-1.1
k__Bacteria	Firmicutes	Erysipelotrichaceae	Bulleidia extructa																																				
k__Bacteria	Firmicutes	Erysipelotrichaceae	Catenibacterium mitsuokai				-0	-0	-0					0		-1	0.02	2.45							-∞	-∞	-∞					∞		-0.5	0.01	0.42			
k__Bacteria	Firmicutes	Erysipelotrichaceae	Coprobacillus bacterium	-0	-0	-0	-0	0.16	-0	0.01	-0	0	0.02	-0	0	0.01		0	-0	-0	-0	-∞	-∞	-∞	-0.4	1.19	-0.3	0.23	-0.4	0.1	0.1	-0.4	0.02	∞		∞	-0.8	-0.3	-0.4
k__Bacteria	Firmicutes	Erysipelotrichaceae	Holdemania filiformis	0.15	0.09	0.07	-0	-0	-0	0.01	-0	0.1	-0	-0.1	-0.1	0.01	0	0.02	0.05	-0.1	-0.1	∞	∞	∞	-1.3	-∞	-∞	0.03	-0.1	0.24	-0.1	-0.3	-0.3	0.23	0.13	0.47	0.19	-0.5	-0.7
k__Bacteria	Firmicutes	Erysipelotrichaceae	Solobacterium moorei			0	0	0.02	-0	-0	-0	-0	0	0			0	0	-0	-0	-0			∞	0.07	0.3	-∞	-0.8	-∞	-∞	∞	∞			∞	∞	-∞	-∞	-0.7
k__Bacteria	Firmicutes	Erysipelotrichaceae	Turicibacter unclassified				0	0.06	0.04				0.01			-0.1	-0	-0	-0	-0	0				∞	∞	∞				∞			-0.6	-0.1	-0.1	-∞	-0.5	0.05
k__Bacteria	Firmicutes	Acidaminococcaceae	Acidaminococcus fermentans													0	0	0																∞	∞	∞			
k__Bacteria	Firmicutes	Veillonellaceae	Dialister invisus				-3.1	-4	-4							3.65									-0.6	-∞	-2							∞					
k__Bacteria	Firmicutes	Veillonellaceae	Dialister microaerophilus								0.01	0.83																	∞	∞									
k__Bacteria	Firmicutes	Veillonellaceae	Megasphaera micronuciformis																																				
k__Bacteria	Firmicutes	Veillonellaceae	Mitsuokella multacida																																				
k__Bacteria	Firmicutes	Veillonellaceae	Selenomonas unclassified			0				0.01	0.01	-0												∞				0.52	0.77	-0.2									
k__Bacteria	Firmicutes	Veillonellaceae	Veillonella atypica					0.17			0.01		0.2	0	0.01		0		0	0.07	-0					∞			∞		1.77	0.03	0.55		∞		0.61	1.95	-∞
k__Bacteria	Firmicutes	Veillonellaceae	Veillonella dispar										0.09		0.01																∞		∞						
k__Bacteria	Firmicutes	Veillonellaceae	Veillonella parvula	0			-0	-0	-0	-0	0.01	-0	0.02	0	0.01							∞			-∞	-∞	-∞	-∞	0.7	-∞	∞	∞	∞						
k__Bacteria	Firmicutes	Veillonellaceae	Veillonella unclassified	0.02		0	-0	-0	0.01	0	0.01	-0	0.1	-0	0.01		0	0	0			∞		∞	-0.5	-∞	0.05	0.03	0.27	-0.6	1.01	-0.3	0.29		∞	∞	∞		
k__Bacteria	Fusobacteria	Fusobacteriaceae	Fusobacterium nucleatum				-0	-0	-0							0.03	-0	-0							-∞	-∞	-∞							0.55	-∞	-∞			
k__Bacteria	Lentisphaerae	Victivallaceae	Victivallis vadensis															0																		∞			
k__Bacteria	Proteobacteria	Brucellaceae	Brucella suis								0.07																		∞										
k__Bacteria	Proteobacteria	Burkholderiaceae	Burkholderia mallei								0.04																		∞										
k__Bacteria	Proteobacteria	Burkholderiaceae	Burkholderia pseudomallei								0.06																		∞										
k__Bacteria	Proteobacteria	Burkholderiaceae	Burkholderia unclassified							-0	-0	0.01									0.02							-∞	-∞	0.44									∞
k__Bacteria	Proteobacteria	Oxalobacteraceae	Oxalobacter formigenes								0.03	0.01	0.01		0.03														∞	∞	∞		∞						
k__Bacteria	Proteobacteria	Sutterellaceae	Sutterella wadsworthensis				-3.8	-4	-3.9							0.4	0.36	0	0.39	1.09	1.43				-1.1	-1.4	-1.2							0.35	0.33	0	0.32	0.6	0.7
k__Bacteria	Proteobacteria	Neisseriaceae	Eikenella corrodens							-0	-0	-0																-0.8	-∞	-∞									
k__Bacteria	Proteobacteria	Neisseriaceae	Neisseria flavescens				-0	-0	-0																-∞	-∞	-∞												
k__Bacteria	Proteobacteria	Neisseriaceae	Neisseria unclassified										0																		∞								
k__Bacteria	Proteobacteria	Desulfovibrionaceae	Bilophila wadsworthia				-0.1	-0.3	-0.3	0.22	0.22	0.87	0.42	0.23	0.66	0.4	-0.3	-0.3		0.03	0.98				-0.3	-∞	-1.5	0.59	0.58	1.08	∞	∞	∞	0.32	-0.6	-1		∞	∞
k__Bacteria	Proteobacteria	Desulfovibrionaceae	Desulfovibrio desulfuricans	0.03	-0	0.01	-0	-0.1	-0.1	0.14	0.09	0.29	0.06	0.03	0.1	0.13	-0.2	-0.2	-0	-0	0.05	1.12	-∞	0.71	-0.2	-∞	-0.9	0.58	0.44	0.84	0.6	0.38	0.78	0.18	-1	-0.9	-0.4	-0.2	0.31
k__Bacteria	Proteobacteria	Desulfovibrionaceae	Desulfovibrio piger				0.01																		∞														
k__Bacteria	Proteobacteria	Enterobacteriaceae	Escherichia coli				1.09	-0.2	-0.6	-1.1	-1.2	-0.8	0.21	-0	0	-0.1	-0.2	1.27	0.23	-0	0.26				0.38	-0.1	-0.7	-0.4	-0.5	-0.3	1.7	-∞	0.12	-0.1	-0.2	0.49	0.84	-0.1	0.89
k__Bacteria	Proteobacteria	Enterobacteriaceae	Escherichia unclassified																																				
k__Bacteria	Proteobacteria	Enterobacteriaceae	Klebsiella pneumoniae																																				
k__Bacteria	Proteobacteria	Enterobacteriaceae	Klebsiella unclassified																																				
k__Bacteria	Proteobacteria	Enterobacteriaceae	Shigella boydii										0.01																		∞								
k__Bacteria	Proteobacteria	Pasteurellaceae	Aggregatibacter aphrophilus				-0	-0	-0			0.01													-1.9	-∞	-∞			∞									
k__Bacteria	Proteobacteria	Pasteurellaceae	Aggregatibacter segnis														0																		∞				
k__Bacteria	Proteobacteria	Pasteurellaceae	Haemophilus parainfluenzae	0.19	0.12	0.03	-0.5	-0.4	-0.7	-0.1	-0	-0.1	0.43	0.04	-0	0.01	0.02	0.01	0.06	-0	-0	1.88	1.71	1.05	-0.5	-0.4	-1	-0.3	-0.1	-0.7	0.91	0.2	-0.7	∞	∞	∞	0.39	-0.2	-∞
k__Bacteria	Proteobacteria	Pseudomonadaceae	Pseudomonas unclassified				0.01			0.01	0		0	0.03		-0	-0	-0							∞			∞	∞		∞	∞		-0.2	-0.5	-∞			
k__Bacteria	Thermi	Deinococcaceae	Deinococcus unclassified	0			0				0		0	0	0.01		0		-0	-0	-0	∞			∞				∞		∞	∞	∞	∞	∞		-0.1	-∞	-0.3
k__Bacteria	Verrucomicrobia	Verrucomicrobiaceae	Akkermansia muciniphila				-0	-0	-0							0.18	0.28	0							-0.2	-∞	-∞							∞	∞	∞			
			6-Point UCDAI	3	2	3	3	4	4	1	1	2	2	2	2	1	5	4	4	4	5	3	2	3	3	4	4	1	1	2	2	2	2	1	5	4	4	4	5
				UCFMT1.R1.S3-2	UCFMT1.R1.S4-2	UCFMT1.R1.S5	UCFMT1.R2.S3-2	UCFMT1.R2.S4	UCFMT1.R2.S5	UCFMT1.R3.S3	UCFMT1.R3.S4	UCFMT1.R3.S5	UCFMT1.R5.S3	UCFMT1.R5.S4	UCFMT1.R5.S5	UCFMT1.R6.S3-2	UCFMT1.R6.S4	UCFMT1.R6.S5	UCFMT1.R7.S3-r	UCFMT1.R7.S4-r	UCFMT1.R7.S5-r	UCFMT1.R1.S3-2	UCFMT1.R1.S4-2	UCFMT1.R1.S5	UCFMT1.R2.S3-2	UCFMT1.R2.S4	UCFMT1.R2.S5	UCFMT1.R3.S3	UCFMT1.R3.S4	UCFMT1.R3.S5	UCFMT1.R5.S3	UCFMT1.R5.S4	UCFMT1.R5.S5	UCFMT1.R6.S3-2	UCFMT1.R6.S4	UCFMT1.R6.S5	UCFMT1.R7.S3-r	UCFMT1.R7.S4-r	UCFMT1.R7.S5-r

Red font: all species that are higher in ulceraticve colitis at baseline.
Blue font: all species that are higher in health at baseline.
Green font: all species that are not detectable at baseline in either UC or healthy samples but that are detected at later timepoints.
Bold Italics: Changes in speces that moved toward the donor.
Regular Font: Changes in species that moved away from the donor.
∞: Change in species that increased from undetectable.
-∞: Change in speces that decreased to undetectable.
Red Numbers: absolute changes of at least 5% and relative changes of at least one logarithm base 10.
####: represents numbers smaller than 0.01
